# Ultrafast Correlation Energy Estimator

**DOI:** 10.1021/acs.jpca.5c04423

**Published:** 2025-09-10

**Authors:** Mateusz Witkowski, Szymon Śmiga, So Hirata, Pavlo O. Dral, Ireneusz Grabowski

**Affiliations:** † Institute of Physics, Faculty of Physics, Astronomy, and Informatics, 414324 Nicolaus Copernicus University in Toruń, ul. Grudzia̧dzka 5, 87-100 Toruń, Poland; ‡ Department of Chemistry, 14589 University of Illinois at Urbana−Champaign, 600 South Mathews Avenue, Urbana, Illinois 61801, United States; § State Key Laboratory of Physical Chemistry of Solid Surfaces, Department of Chemistry, College of Chemistry and Chemical Engineering, and Fujian Provincial Key Laboratory of Theoretical and Computational Chemistry, 12466 Xiamen University, Xiamen 361005, China; ∥ Aitomistic, Shenzhen 518000, China

## Abstract

A virtually no-cost method is proposed that can compute the correlation energies of general, covalently bonded, organic, and inorganic molecules (including conjugated π-electron systems) with a well-defined dominant Lewis structure at the accuracy of 99.5% of the near-exact values determined by the coupled-cluster singles, doubles, and perturbative triples [CCSD­(T)] in the complete-basis-set (CBS) limit. This Correlation Energy Per Bond (CEPB) method assigns a partial correlation energy to each bond type (characterized by the identities of the two atoms forming the bond and its integer bond order) and to a lone pair, regardless of the bond length, bond angle, sp-hybridization, π-electron conjugation, ionicity, noncovalent interactions, etc. At its current stage, the method is mainly suitable for near-equilibrium geometries. The correlation energies per bond are determined by a fit to the CCSD­(T)/CBS benchmarks. It can neither improve the equilibrium structures nor discern conformers or positional isomers, yet its accuracy for reaction energies rivals that of the second-order Møller–Plesset perturbation theory, which is far more expensive. Its promising performance underscores the possibility that surprisingly compact, chemically intuitive molecular fragments exist into which correlation energies can be partitioned, leading to various ultrafast correlation-energy estimators tailored to different purposes.

## Graphical abstract

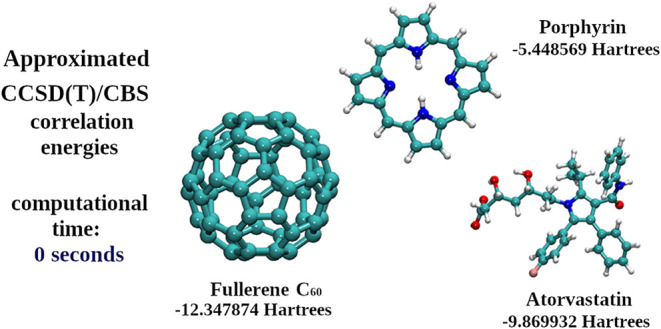

## Introduction

While *ab initio* Hartree–Fock (HF) theory in a large basis set can recover the total electronic energy of a “well-behaved” molecule (i.e., having a dominant Lewis valence-bond structure) typically 99.5% of the exact value, the vast majority of it comes from the atomic core electrons, and accurate descriptions of its structure, properties, and reactivities still require inclusion of electron correlation among valence electrons. Of many correlation methods, the coupled-cluster (CC) method with singles, doubles, and perturbative triples [CCSD­(T)] ^1,2^ is considered the most accurate for a reasonable cost and is frequently referred to as the “gold standard” of modern computational chemistry. Nevertheless, its cost increases as the staggering seventh power of system size, and its applicability to larger molecules is still severely limited.

A broad class of fragmentation methods has been developed to address the cost issue.^3,4^ It is the most aggressive type of approach based on the notion of the “near-sightedness” of electron-correlation effects,^5^ which thus can be described as accumulated independent effects, each localized to a small spatial region of a very large system.^6,7^ In these approaches, therefore, a large molecule, solid^8,9^ or liquid^10^ is partitioned into small fragments, whose correlation or total energies are evaluated separately (often in parallel) and then summed over to produce the whole system’s total (correlation) energy. They calculate interaction energies between overlapping or neighboring fragments, constituting the total energy.

Examples include the Fragment Molecular Orbital (FMO) method^11–13^ and Electrostatically Embedded Generalized Molecular Fractionation with Conjugate Caps (EE-GMFCC),^14^ primarily designed for the total energies of polypeptides and proteins. The Generalized Energy-Based Fragmentation (GEBF)^15,16^ and Molecular Tailoring Approach (MTA)^17–19^ use spheres with a given radius to divide large molecules. The Systematic Molecular Fragmentation (SMF)^20,21^ and the Combined Fragmentation Method (CFM),^22^ on the other hand, rely on more chemically intuitive concepts such as bonds and functional groups for fragmentation. Some methods, such as the Many-Body Expansion (MBE),^23^ partition a system into subsystems with increasing size, starting from including just fragment monomers, followed by dimers, trimers, and so on.^24^

A molecular system can be partitioned at a more microscopic level, such as at the levels of atoms or localized orbitals. The pioneering work of the local correlation approach by Saebo and Pulay^6^ used localized orbitals as the partitioning basis. Perhaps the most advanced instance of this approach is the LNO–CCSD­(T) method.^25–28^ It dramatically broadens the applicability of the CCSD­(T) method to larger systems of any type with almost no loss of accuracy in most cases. Similar principles apply to PNO–CCSD­(T),^29^ DLPNO–CCSD­(T),^30^ and others.^31,32^

More recently, a whole new path to fast electron-correlation methods has emerged, exploiting the machine learning (ML) and artificial intelligence (AI), which are rapidly advancing chemical computations today.^33–40^ Their ability to identify hidden patterns in molecular and electronic structures can be harnessed to reach an unusual but practical method with a dramatically reduced computational cost. These methods are reshaping quantum chemical simulations, as the development of traditional quantum mechanical methods often requires substantial resources while offering only modest improvements. Considerable effort is put into developing such ML models, which can produce the CCSD­(T)-quality results at a fraction of the cost. Some of these approaches predict the correlation energy, e.g., MOB-ML^41^ and data-driven CCSD,^42^ or the total CCSD­(T) energy directly, e.g., the ANI-1ccx^43^ and ANI-1ccx-gelu^44^ potentials and Δ-learning-based^45^ AI-enhanced quantum mechanical methods AIQM.^46,47^ Particularly, the latter have demonstrated a broad range of applicability, achieving accuracy comparable to the CC level, while their numerical cost is relatively small compared to *ab initio* or density functional theory (DFT) methods.

Although these new methods show promising results, the abundance of parameters used in training models can somewhat hinder our understanding of the problem. Indeed, we gain access to “black box” machinery that can outperform the best standard methods, but in doing so, we may perceive a sacrifice in interpretability.^48^ Therefore, identifying the most crucial molecular descriptors relevant to specific properties should remain a vital aspect of current research.

These methods describe molecular systems without using such concepts as chemical bonding. However, Bader has shown^49,50^ that chemical bonding can easily be introduced by utilizing electron density analysis. It connects the “standard” chemistry within the molecule, which can be presented as a drawing with lines (symbolizing chemical bonds) between atoms, with the sophisticated description of molecules resulting from the solution of the Schrödinger equation.

In this context, we have developed a novel approach to address the scaling problem in correlation energy calculations. Our method utilizes only the minimally necessary variables, focusing specifically on those critical to the correlation energy. In some respects, it follows energy-based molecular fragmentation strategies, where a large molecule is divided into relatively small fragments. The sum of these fragments’ correlation energies approximates the entire system’s total correlation energy. Specifically, our methodology decomposes the molecular correlation energy into smaller, localized contributionsnamely, the correlation energies associated with individual chemical bonds. We remark that Grassi et al.^51^ have also attributed the correlation energy to chemical bonds. However, they included additional terms of the correlation energy per atom, which led to the physically misleading concept of the correlation energy of a single hydrogen atom. Furthermore, their approach requires computing bond order according to Löwdin formulation,^52^ whereas we rely on a more straightforward classification of bonds into single, double, and triple bond categories.

We assume the transferability of the values of those parts of the correlation energies between different molecular systems, and therefore, the correlation energies per bond can be estimated once from independent calculations, e.g., CCSD­(T) or even FCI ones, and then used for any molecular system to calculate the total correlation energy.

The new approach, Correlation Energy Per Bond (CEPB), uses the CCSD­(T) method in our case to calculate correlation energies per bond, and our CEPB results approximate the CCSD­(T) correlation energies. It is worth underlining that after obtaining the correlation energies per bond values, the computational time for calculating correlation energies for arbitrarily large molecular systems is negligible. At the current stage of development, bond-length dependence is not yet incorporated in the procedure. Therefore, the most promising results are obtained for molecules near their equilibrium geometries. The inclusion of bond-length dependence will be addressed in future work. In this paper, we present the core concept that forms the foundation of the new method.

## Theory and Methodology

To establish our methodology, we adopt the following assumptions:

- The correlation energy of a neutral molecular system can be expressed as the sum of bond-specific correlation energies and the contributions from nonbonding lone pairs of electrons (LPEs) occurring in this molecule. Here, we follow the general understanding of a chemical bond as a pair(s) of electrons shared by two neighboring atoms. Our method is based on the simple yet powerful assumption that each chemical bond within a molecule contributes a distinct portion of the total correlation energy, and the sum of these contributions accurately reproduces the molecules overall correlation energy.
  To justify this assertion, we consider the carbon dioxide (CO_2_) molecule and compute its correlation energy using the CCSD­(T) method in conjunction with the aug-cc-pVQZ basis set. If the correlation energy were entirely independent of chemical bonding, it would be feasible to approximate the total molecular correlation energy by summing the correlation energies of the constituent atoms. However, in our case, the correlation energy of CO_2_ is equal to −0.759919 *E*_h_, while the sum of contributions from carbon atom (−0.119145 *E*_h_) and two oxygen atoms (−0.207327 *E*_h_ each) correlation energies is equal to −0.533799 *E*_h_ (see Table 1). This significant difference must be inherently linked to the formation of chemical bonds.
- We deliberately exclude atomic contributions. Previous studies have demonstrated that the correlation energy’s valence–valence (VV) component constitutes the most significant portion, accounting for approximately 70% of the total correlation energy in systems such as the oxygen atom.^54^ This supports our decision, as valence-shell electrons are directly involved in chemical bonding.^54^ Furthermore, the correlation effects associated with core electrons are expected to remain largely unaffected by bond formation. We therefore assume that their contributions to the correlation energy are effectively redistributed across the bonded regions. In this way, core electron correlation is indirectly included, allowing us to simplify the methodology without sacrificing accuracy. Recently, Martin^55^ presented an analysis of the correlation energy densities based on Chachiyo’s correlation energy density functional.^56^ The study revealed that the correlation energy density between atoms is significantly affected during the formation of chemical bonds, offering additional support for the reliability of our CEPB method.
- The key assumption in our work is the transferability of the correlation energy per bond quantities (for a fixed two-atomic pair and bond type) between different molecular systems. As will become apparent in the later sections of this paper, this assumption is well justified.

**Table 1:** Correlation Energy Contributions for CO_2_ Molecule at CCSD­(T)/aug-cc-pVQZ Level^a^

component	correlation energy (*E*_h_)
carbon (C)	–0.119145
oxygen (O) (each)	–0.207327
sum of atomic contributions (C + 2O)	–0.533799
total correlation energy of CO_2_	–0.759919
difference (bonding effect)	–0.226120

a The correlation energies were taken from the NIST Database.^53^

In practice, we use a given molecule’s Lewis structure^57^ to identify and count different types of chemical bonds together with the lone pairs of electrons. Then we assign the correlation energy for a specific bond type, e.g., carbon–hydrogen bond C–H or carbon–carbon: single C–C, double C=C, and triple C≡C bonds separately. Note that in the following we use a simplified notation of the correlation energies per bond, e.g., $E_{C-H}^{\mathrm{corr}}=\left[C-H\right]$ . The complete list of bond types used in this paper is presented in Table 2. We also treat the LPE as an additional bond type and assign it a correlation energy [LPE].

**Table 2:** Correlation Energies Per Bond Values $E_{j}^{\mathrm{corr}}$ (in *E*_h_) of 33 Different Chemical Bonds Obtained at CCSD­(T) Level for aug-cc-pVTZ, aug-cc-pVQZ Basis Sets, and CBS Limit^a^

	$E_{j}^{\mathrm{corr}}$ (*E*_h_)			
bond type	aug-cc-pVTZ	aug-cc-pVQZ	CBS	aug-cc-pVTZ (fc)
[LPE]	–0.066851	–0.078726	–0.087392	–0.058023
[C–C]	–0.088570	–0.097015	–0.103179	–0.079455
[C–H]	–0.060762	–0.065666	–0.069244	–0.055798
[C=C]	–0.173583	–0.191884	–0.205239	–0.156073
[C≡C]	–0.251915	–0.280422	–0.301224	–0.226291
[C=O]	–0.205731	–0.222163	–0.234153	–0.199584
[C≡N]	–0.276598	–0.303309	–0.322800	–0.256715
[O–H]	–0.080848	–0.085117	–0.088233	–0.082043
[N–H]	–0.069244	–0.074040	–0.077540	–0.066513
[S=O]	–0.209657	–0.229257	–0.243559	–0.198993
[C–S]	–0.098155	–0.106985	–0.113429	–0.082407
[S–H]	–0.063518	–0.068887	–0.072805	–0.052862
[C–O]	–0.108968	–0.116852	–0.122605	–0.104800
[C–Br]	–0.147279	–0.213122	–0.261170	–0.121355
[C–Cl]	–0.100828	–0.114334	–0.124190	–0.087435
[C–F]	–0.122442	–0.129309	–0.134321	–0.129913
[C=S]	–0.185576	–0.205400	–0.219867	–0.157709
[S–Cl]	–0.102715	–0.118182	–0.129468	–0.085560
[C–N]	–0.100471	–0.108552	–0.114449	–0.092186
[N–O]	–0.119241	–0.126550	–0.131884	–0.118044
[C=N]	–0.191964	–0.209382	–0.222093	–0.177105
[S–O]	–0.096424	–0.105302	–0.111781	–0.090401
[P–P]	–0.097144	–0.098537	–0.099554	–0.070260
[P–Cl]	–0.099488	–0.110806	–0.119064	–0.078065
[P–H]	–0.062889	–0.064127	–0.065031	–0.049592
[P=O]	–0.196635	–0.216180	–0.230443	–0.187626
[P–O]	–0.105588	–0.110941	–0.114846	–0.092653
[P–F]	–0.125826	–0.129211	–0.131681	–0.124472
[O–O]	–0.135303	–0.142152	–0.147149	–0.138497
[S–S]	–0.103754	–0.113499	–0.120611	–0.083480
[N–N]	–0.107878	–0.115556	–0.121159	–0.102220
[N=O]	–0.233831	–0.248844	–0.259800	–0.231316
[C–P]	–0.096921	–0.101224	–0.104364	–0.077885

a The last column presents the frozen-core (fc) CCSD­(T)/aug-cc-pVTZ results. The [LPE] “bond type” assigns the correlation energy per one lone pair of electrons.

Based on this approach, we calculate the correlation energy of a given molecule from a general expression

1 $$E_{X}^{\mathrm{corr}}=\sum_{j=1}n_{j}^{X}E_{j}^{\mathrm{corr}}$$

where *E*_*X*_^corr^ is correlation energy of the *X* molecule, *n*_*j*_^*X*^ is the number of *j*–th type chemical bonds in the *X* molecule, and $E_{j}^{\mathrm{corr}}$ is the correlation energy assigned to *j*–th type of chemical bond, including [LPE] “bond type”.

To calculate correlation energies using this method, one only needs to know the types and numbers of chemical bonds in the given molecular system and their associated correlation energies per bond values. To illustrate in practice this method, we compute the correlation energy of a few species with increasing complexity. For example, the correlation energy of methane (CH_4_) can be expressed by the CEPB method as the sum of four [C–H] bond contributions as follows

$$E_{{\mathrm{CH}}_{4}}^{\mathrm{corr}}=4E_{C-H}^{\mathrm{corr}}=4\left[C-H\right]$$ 2

Next, as a more illustrative example, we consider the calculation of the correlation energy of the formaldehyde (H_2_CO) molecule. It consists of two hydrogens, one carbon, and one oxygen atom, which together have 12 valence electrons. The octet rule distributes these electrons into chemical bonds and LPE as shown in Figure 1.

**Figure 1:**
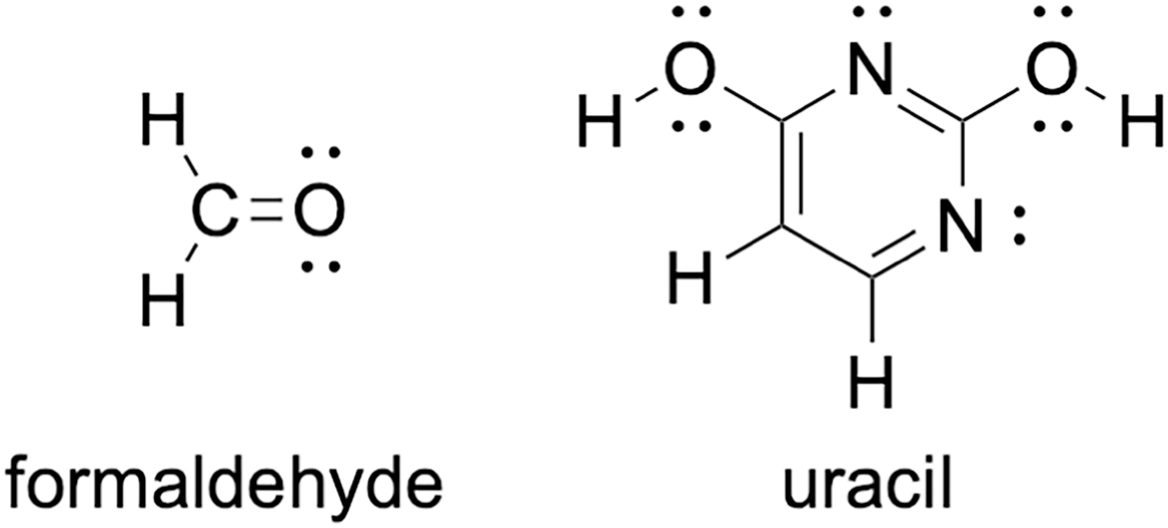
Lewis structures of formaldehyde and uracil (lactim form).

According to eq 1, the correlation energy of formaldehyde can be written as

$$E_{H_{2}\mathrm{CO}}^{\mathrm{corr}}=2\left[\mathrm{LPE}\right]+2\left[C-H\right]+\left[C=O\right]$$ 3

where [LPE] is the correlation energy per one lone pair of electrons, and [C–H] and [C=O] are the correlation energies per single carbon–hydrogen and double carbon–oxygen bond, respectively. Again, the formula in eq 3 is consistent with the notation of eq 1, i.e., $E_{H_{2}\mathrm{CO}}^{\mathrm{corr}}=2E_{\mathrm{LPE}}^{\mathrm{corr}}+2E_{C-H}^{\mathrm{corr}}+E_{C=O}^{\mathrm{corr}}$ .

As a final example, we present the uracil molecule (C_4_N_2_O_2_H_4_, lactim form), one of the systems presented in Table 4, with its Lewis structure (see Figure 1).

In this case, the correlation energy of the CEPB method can be written as

$$E_{C_{4}N_{2}O_{2}H_{4}}^{\mathrm{corr}}=6\left[\mathrm{LPE}\right]+2\left[C-H\right]+2\left[C-O\right]+\left[C-C\right]+\left[C=C\right]+2\left[C=N\right]+2\left[C-N\right]+2\left[O-H\right]$$ 4

In such a way, it is possible to calculate the correlation energies of almost any neutral molecule if one knows the number and type of chemical bonds in this molecule and the values of the correlation energies per specific type of bonds $E_{j}^{\mathrm{corr}}$ .

To make a method feasible, one needs to find how to calculate the values of the correlation energies per bond $E_{j}^{\mathrm{corr}}$ . For some types of bonds, it can be easily estimated. For example, the correlation energy per the single C–H bond, [C–H], can be directly calculated, e.g., from the methane CH_4_ correlation energy (see eq 2). We can use any “reasonable” CH_4_ correlation energy, e.g., from second-order Møller–Plesset (MP2)^58^ or CCSD­(T) calculations. Our example uses correlation energy calculated from the CCSD­(T) method in the Complete Basis Set (CBS) limit. For this case, the correlation energy per single carbon–hydrogen bond equals [C–H] = −0.070116 *E*_h_.

Now, using the [C–H] value, and CCSD­(T) ethylene C_2_H_4_ correlation energy, we can calculate the correlation energy per double carbon–carbon bond [C=C] directly from eq 5 as

$$E_{C_{2}H_{4}}^{\mathrm{corr}}=4\left[C-H\right]+\left[C=C\right]$$ 5

from which [C=C] equals −0.191197 *E*_h_. Similarly, we can calculate a single [C–C] and triple [C≡C] bond correlation energies from the CCSD­(T) correlation energies of C_2_H_6_ and C_2_H_2_ molecules and eqs 6 and 7, respectively:

$$E_{C_{2}H_{6}}^{\mathrm{corr}}=6\left[C-H\right]+\left[C-C\right]$$ 6

$$E_{C_{2}H_{2}}^{\mathrm{corr}}=2\left[C-H\right]+\left[C\equiv C\right]$$ 7

From the above, we will have [C–C] of −0.103477 *E*_h_ and [C≡C] of –0.303466 *E*_h_ correlation energies per single and triple carbon–carbon bond, respectively. We can continue this procedure and calculate correlation energies for other bond types step by step using previously calculated values or solving simple sets of linear equations. However, this way of proceeding is ineffective and may produce different results for different choices of the “starting point,” i.e., the first type of bond calculated, the selection of subsequent molecules, and the order of including other types of bonds and calculating their correlation energy per bond.

Therefore, we propose a much more effective way of calculating the correlation energies for a specific bond type.

- First, we define a set of different types of chemical bonds, collected in Table 2, that can be useful or essential for calculating the correlation energy of larger systems. We add the [LPE] “bond type” to our set of bond types to assign the correlation energy per one lone pair of electrons. This set (Table 2) can be extended to other bond types if necessary.
- Then we select the training set of molecules, in which such bonds occur and for which we can relatively efficiently compute the CCSD­(T) correlation energies (see Table 3). Every type of bond is present in at least three different molecules from the set to ensure the most reliable results. This set can be expanded in the future.
- For each molecule from the training set, using the Lewis structures, we represent their correlation energy by the bond correlation energies $E_{j}^{\mathrm{corr}}$ according to eq 1. In this formula, the number of different types of bonds *n*_*j*_^*X*^ is fixed (known) for each molecule, and we can “optimize” the correlation energies per bond $E_{j}^{\mathrm{corr}}$ to reproduce the CCSD­(T) correlation energies for all molecules in the training set.
- We perform the optimization of $E_{j}^{\mathrm{corr}}$ using the standard multiple linear regression method, which can be achieved by minimizing the loss function L
8 $$L=\sum_{i}{\left(E_{i}^{\mathrm{corr},\mathrm{ref}}-\sum_{j=1}n_{j}^{i}E_{j}^{\mathrm{corr}}\right)}^{2}$$
  with respect to $E_{j}^{\mathrm{corr}}$ . In the eq 8, the *i* runs over all molecules from the training set; *E*_*i*_^corr,ref^ is the reference correlation energy of the *i*–th molecule calculated on a chosen level of theory (MP2, CCSD­(T), even FCI). In this work *E*_*i*_^corr,ref^ has been obtained from the CCSD­(T)/CBS calculations. The final values of optimized correlation energies per bond $E_{j}^{\mathrm{corr}}$ for several types of bonds are presented in Table 2. The step-by-step procedure of obtaining correlation energies per bond is also presented on the Figure 2.
- Finally, using the Lewis structure analysis together with eq 1 and the calculated optimal values of correlation energies for specific bonds $E_{j}^{\mathrm{corr}}$ collected in Table 2, we can calculate correlation energy of any neutral molecule at its equilibrium geometry. The step-by-step procedure is presented on the Figure 3. The atomic connectivity, especially for large molecules, can be easily obtained from a Structural Data File (SDF), e.g., from PubChem.^59^ We implemented a custom script that parses SDF files to extract molecular connectivity information and automatically generates a list of bond types and their counts for a given molecule. Then, the CEPB correlation energies of that molecule are printed for the basis sets presented in Table 2. The script is available from the corresponding author upon reasonable request.

**Figure 2:**
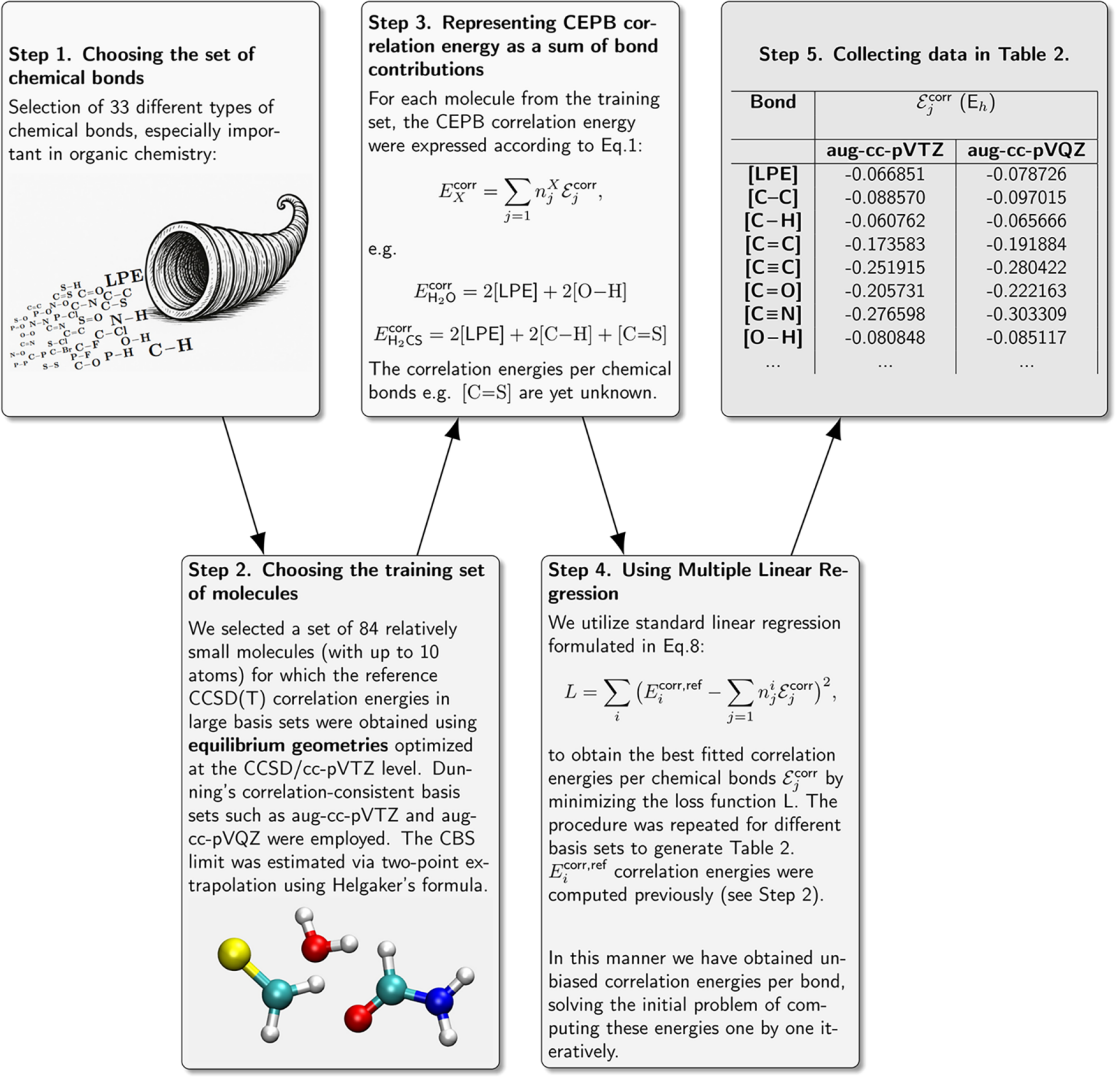
Procedure for obtaining the unbiased correlation energies per chemical bond. The visual weight (e.g., font size or line thickness) assigned to each bond in the figure in Step 1 is proportional to its frequency of occurrence in the training set of molecules. The graphic in Step 1 was produced with partial assistance from an AI-based image generator.

**Figure 3:**
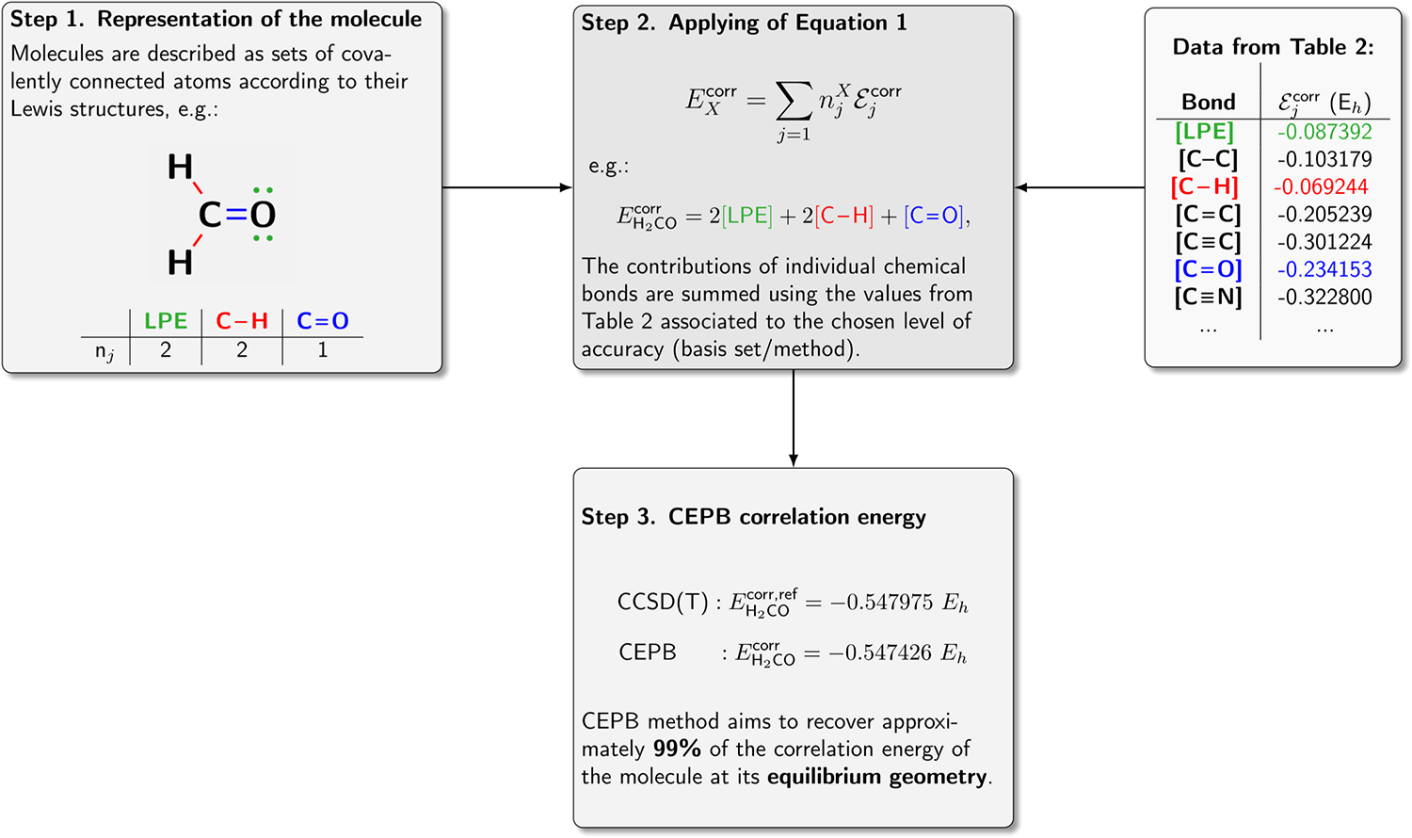
Step-by-step scheme illustrating the procedure for obtaining the CEPB correlation energy at a specified level of accuracy. Once Table 2 is constructed, the only cost of the CEPB method is to count the number of different chemical bonds. This task can also be automated using a Structural Data File as input to our custom script, available from the corresponding author.

**Table 3:** Correlation Energies for 84 Molecules (in *E*_h_) from the Training Set Calculated Using CCSD­(T), CCSD, MP2, and CEPB Methods in the CBS Limit^a^

		*E*_*i*_^corr^ (*E*_h_)		error (%)		
compound		CCSD(T)	CEPB	CEPB	CCSD	MP2
methane	CH_4_	–**0.280463**	–0.276977	1.24	2.74	8.87
ethane	C_2_H_6_	–**0.524171**	–0.518643	1.05	3.11	8.05
ethene	C_2_H_4_	–**0.481912**	–0.482215	0.06	3.72	7.80
acetylene	C_2_H_2_	–**0.443698**	–0.439712	0.90	4.37	6.18
formaldehyde	H_2_CO	–**0.547975**	–0.547426	0.10	3.70	4.60
carbon monoxide	CO	–**0.496609**	–0.496330	0.06	4.10	3.87
carbon dioxide	CO_2_	–**0.817453**	–0.817875	0.05	4.18	2.28
acetonitrile	CH_3_CN	–**0.721511**	–0.721103	0.06	4.13	5.18
water	H_2_O	–**0.352771**	–0.351250	0.43	2.91	3.35
ammonia	NH_3_	–**0.321980**	–0.320011	0.61	3.03	5.65
sulfur dioxide	SO_2_	–**0.935716**	–0.924080	1.24	4.59	2.64
sulfur trioxide	SO_3_	–**1.249351**	–1.255031	0.45	4.38	2.25
thiirane	C_2_H_4_S	–**0.779352**	–0.781797	0.31	4.19	6.69
methanethiol	CH_3_SH	–**0.568675**	–0.568751	0.01	3.69	8.14
formic acid	HCOOH	–**0.861820**	–0.863805	0.23	3.74	3.40
ethanethiol	C_2_H_5_SH	–**0.814597**	–0.810417	0.51	3.70	7.70
2–mercaptoethanol	C_2_H_6_OS	–**1.127624**	–1.126796	0.07	3.68	6.09
bromomethane	CH_3_Br	–**0.729325**	–0.731079	0.24	3.34	3.27
chloromethane	CH_3_Cl	–**0.591312**	–0.594099	0.47	3.60	7.45
dichloromethane	CH_2_Cl_2_	–**0.906588**	–0.911221	0.51	3.96	6.84
trichloromethane	CHCl_3_	–**1.225752**	–1.228344	0.21	4.19	6.42
carbon tetrachloride	CCl_4_	–**1.548355**	–1.545466	0.19	4.37	6.09
fluoromethane	CH_3_F	–**0.605528**	–0.604230	0.21	2.84	4.68
difluoromethane	CH_2_F_2_	–**0.932245**	–0.931483	0.08	2.91	3.35
trifluoromethane	CHF_3_	–**1.259163**	–1.258736	0.03	2.95	2.70
carbon tetrafluoride	CF_4_	–**1.584963**	–1.585989	0.06	2.96	2.35
methanol	CH_3_OH	–**0.592221**	–0.593355	0.19	3.15	5.26
ethanol	C_2_H_5_OH	–**0.837401**	–0.835022	0.28	3.30	5.74
thioformaldehyde	H_2_CS	–**0.530519**	–0.533139	0.49	4.48	7.65
hydrogen cyanide	HCN	–**0.479640**	–0.479437	0.04	4.42	4.32
carbonyl sulfide	OCS	–**0.802644**	–0.803589	0.12	4.82	3.99
carbon disulfide	CS_2_	–**0.791466**	–0.789302	0.27	5.61	5.43
thionyl chloride	SOCl_2_	–**1.291557**	–1.289026	0.20	4.63	5.23
phosgene	COCl_2_	–**1.184223**	–1.181671	0.22	4.33	4.99
formyl chloride	CHClO	–**0.865251**	–0.864548	0.08	4.14	4.91
thiocyanic acid	HSCN	–**0.770599**	–0.771211	0.08	4.60	5.33
isothiocyanic acid	HNCS	–**0.776888**	–0.781675	0.62	4.98	4.89
dimethyl ether	CH_3_OCH_3_	–**0.834832**	–0.835460	0.08	3.31	5.93
formamide	HCONH_2_	–**0.832325**	–0.835102	0.33	3.80	4.11
methylamine	CH_3_NH_2_	–**0.563830**	–0.564653	0.15	3.24	6.45
hydroxylamine	NH_2_OH	–**0.636962**	–0.637373	0.06	3.40	4.30
methanimine	CH_2_NH	–**0.522835**	–0.525513	0.51	3.85	5.88
ethenamine	CH_2_CHNH_2_	–**0.767596**	–0.769891	0.30	3.81	6.15
thioacetaldehyde	CH_3_CHS	–**0.775474**	–0.774806	0.09	4.25	7.26
ethenethiol	CH_2_CHSH	–**0.774419**	–0.773989	0.06	4.17	7.40
cyclopropene	C_3_H_4_	–**0.692103**	–0.688572	0.51	4.15	6.36
cyclopropenylidiene	C_3_H_2_	–**0.629400**	–0.637476	1.28	4.41	6.47
cyclopropyne	C_3_H_2_	–**0.639615**	–0.646070	1.01	4.99	6.68
sulfur dichloride	SCl_2_	–**0.956594**	–0.958074	0.15	4.40	7.11
methoxyamine	CH_3_ONH_2_	–**0.878973**	–0.879478	0.06	3.48	5.21
*N*–methylhydroxylamine	CH_3_NHOH	–**0.880573**	–0.882015	0.16	3.49	5.10
chlorosulfuric acid	HSO_3_Cl	–**1.601027**	–1.603130	0.13	4.14	3.46
phosphorus trichloride	PCl_3_	–**1.230572**	–1.231115	0.04	4.40	6.78
phosphine	PH_3_	–**0.283593**	–0.282484	0.39	3.38	11.53
hypophosphorous acid	H_3_PO_2_	–**0.912337**	–0.913152	0.09	3.59	3.99
phosphoryl chloride	POCl_3_	–**1.549494**	–1.548950	0.04	4.30	5.33
diphosphane	P_2_H_4_	–**0.534659**	–0.534461	0.04	4.04	10.42
orthophosphoric acid	H_3_PO_4_	–**1.539090**	–1.538818	0.02	3.56	2.96
phosphorus trifluoride	PF_3_	–**1.269044**	–1.268966	0.01	3.10	2.78
diphosphorus tetrafluoride	P_2_F_4_	–**1.849711**	–1.849770	0.00	3.34	3.37
diaminomethane	NH_2_CH_2_NH_2_	–**0.849578**	–0.852329	0.32	3.48	5.56
hydrazine	N_2_H_4_	–**0.607160**	–0.606101	0.17	3.46	5.29
hydrogen peroxide	H_2_O_2_	–**0.671906**	–0.673184	0.19	3.48	3.53
1,1–dimethylhydrazine	H_2_N_2_(CH_3_)_2_	–**1.096596**	–1.095385	0.11	3.59	5.99
oxirane	H_2_COCH_2_	–**0.799058**	–0.800151	0.14	3.69	5.05
methyl hydroperoxide	H_3_CO_2_H	–**0.914208**	–0.915289	0.12	3.56	4.59
hydroperoxyamine	H_2_NO_2_H	–**0.961664**	–0.959306	0.25	3.80	3.96
methyldisulfide	H_3_CS_2_H	–**0.861733**	–0.864146	0.28	4.15	7.74
bromoethane	C2H_5_Br	–**0.976411**	–0.972746	0.38	3.44	4.10
dibromomethane	CH_2_Br_2_	–**1.184226**	–1.185181	0.08	3.56	1.77
ethanimine	C_2_H_5_N	–**0.765880**	–0.767180	0.17	3.80	6.29
dimethyldisulfide	C_2_H_6_S_2_	–**1.109369**	–1.112502	0.28	4.12	7.41
methyl–disphosphin	CH_6_P_2_	–**0.781388**	–0.781527	0.02	3.99	9.12
methylphosphine	CH_5_P	–**0.528515**	–0.529550	0.20	3.54	9.22
propargylchloroformate	C_4_H_3_ClO_2_	–**1.829905**	–1.827435	0.13	4.24	4.56
nitrosamine	H_2_N_2_O	–**0.883314**	–0.885607	0.26	4.22	3.70
trimethylphosphine	C_3_H_9_P	–**1.024074**	–1.023682	0.04	3.70	7.79
thioureadioxide	CH_4_N_2_O_2_S	–**1.746672**	–1.737908	0.50	4.24	3.91
methylhydrazine	CH_3_NHNH_2_	–**0.849137**	–0.850743	0.19	3.49	5.78
disulfane	H_2_S_2_	–**0.614621**	–0.615790	0.19	4.22	8.31
methanesulfonicacid	CH_4_O_3_S	–**1.525986**	–1.532647	0.44	3.89	3.60
nitrosodimethylamine	(CH_3_)_2_NN=O	–**1.376520**	–1.374890	0.12	4.11	4.77
thiothiophthene	C_5_H_4_S_3_	–**2.022074**	–2.018717	0.17	5.46	5.30
nitroxyl	HNO	–**0.600180**	–0.599516	0.11	4.23	4.20
			**MAE**	0.002	0.035	0.046
			**MAPE**	0.27	3.88	5.48
			**RMS**	0.003	0.039	0.050

a The last three columns show the percentage errors with respect to CCSD­(T) results. The MAE, RMS (in *E*_h_), and MAPE (in %) are reported in the last rows.

## Computational Details

In this work, we selected 33 different types of bonds (including LPE) listed in Table 2, which cover most of the bonds essential in organic chemistry. Then, we constructed the training set of 84 molecules and calculated their CCSD­(T) correlation energies. All calculations have been performed using PySCF^60^ and MRCC^61,62^ software packages. If not otherwise stated, all electrons were included in the calculations. To allow a comparison of our method with results available in the literature for larger systems, we have also performed CCSD­(T) calculations on an aug-cc-pVTZ basis with the frozen core. We utilized the geometries optimized at the CCSD/cc-pVTZ level for the molecules in the training set. A complete basis set (CBS) limit was employed for correlation energies using Helgaker’s two-point formula^63,64^ based on aug-cc-pVTZ and aug-cc-pVQZ calculations. As an approximation to the CBS limit of HF energy, the aug-cc-pV5Z results were utilized. Additionally, several calculations for very big systems were performed with the UAIQM methods^46,65^ (versions 20240106 aka AIQM2 ^47^ and 20250115) as implemented in MLatom^66^ with Aitomic^67^ add-ons. UAIQM calculations required MLatom’s interface to the TorchANI^68^ package providing the neural-network Δ-learning corrections^45^ to the GFN2-xTB* ^65,69^ baseline energies calculated via the interface to the xtb program^70^ program; in addition, the calculations include the D4-dispersion corrections via MLatom’s interface to the DFTD4 program.^71,72^ All presented energies are reported in *E*_h_ unless otherwise specified.

The correlation energies per bond were obtained by solving the optimization problem given by eq 8. The corresponding values are reported in Table 2 for aug-cc-pVTZ, aug-cc-pVQZ basis sets, and at the CBS limit. In addition, we calculated correlation energies per bond for a frozen core aug-cc-pVTZ CCSD­(T) (last column in Table 2), which allows us to make some useful comparisons with literature results.

## Results and Discussion

The energies from Table 2 are directly used to calculate the CEPB correlation energies of all systems presented in this paper. We can notice that the values of correlation energies per bond, i.e., [C–H], [C=C], [C–C], and [C≡C], which are calculated directly from eqs 2, 5, 6, and 7 are qualitatively similar to the final optimal values presented in Table 2 with errors around 1% in most cases. The largest difference (≈7%) is visible for the case of [C=C] CEPB energy.

First, we analyze the values of the correlation energies per bond, which are reported in Table 2. One can note that the absolute value of the correlation energy per bond grows with the increasing bond multiplicity. This is additionally visually presented in Figure 4. Despite one exception noted for the carbon–bromine bond, the correlation energies per bond are qualitatively similar within single, double, and triple bonds.

**Figure 4:**
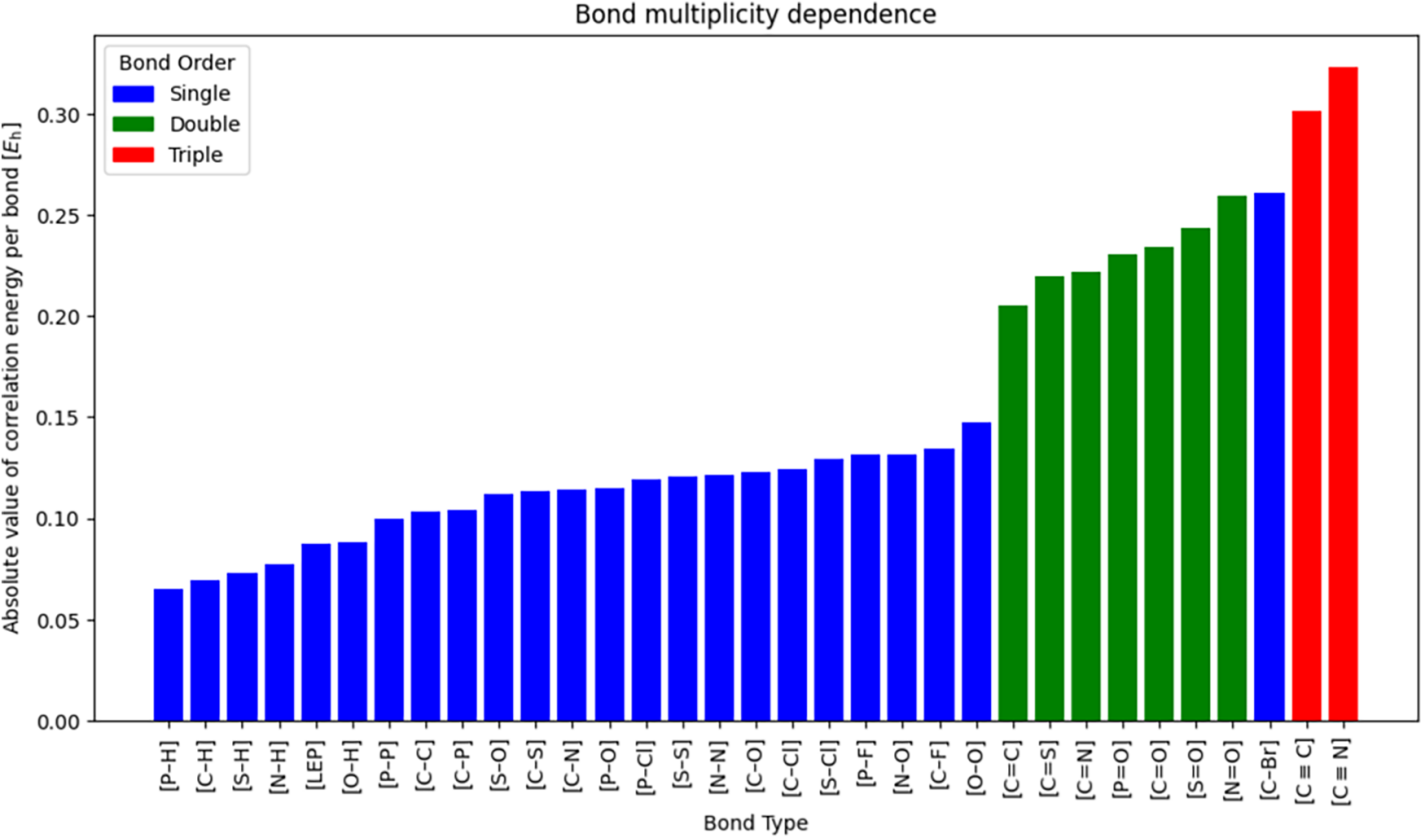
Absolute values of correlation energies for chemical bonds from Table 2 at the CCSD­(T)/CBS limit level.

These apparent similarities, upon closer inspection, disclose rather intriguing behavior. For instance, the absolute value of the CEPB of the P–H bond is smaller than that for the N–H bond, which can be considered an effect related to the period, as phosphorus is positioned below nitrogen in the periodic table. We have found that this pattern holds in all possible cases: the absolute value of CEPB of the S–H bond is lower than O–H (since sulfur is below oxygen), P–P is lower than N–N, C–S is lower than C–O, C–Cl is lower than C–F (chlorine is positioned below fluorine), P–Cl is lower than P–F, and so on. The reader can verify other bond pairs, such as C–P versus C–N and P–O versus N–O, and homonuclear pairs, such as S–S versus O–O. Interestingly, even for double bonds, we can recognize this effect for C=S versus C=O or P=O versus N=O pairs.

The decreasing absolute value of CEPB, after replacing the second-row element of the periodic table with its alternative from the third row, seems to be related to the size of atoms. It is well-known that covalent radii decrease across periods (left to right) and increase down groups in the periodic table.^73^ Suppose that the atomic size is genuinely related to the observed phenomena. In that case, a similar effect should also be noticeable if we replace one atom in the bond with the element positioned to the right in the periodic table. Since the atomic radius decreases with increasing group number, the absolute value of CEPB should also increase after the replacement, e.g., carbon by nitrogen. Indeed, the value for the C–H bond is lower than that of N–H, which is lower than that of O–H. Similarly, homonuclear pairs C–C and N–N also hold (in this case, both carbon atoms were replaced). We can also observe it for other atomic pairs, e.g., C–O is lower than N–O, C–C versus C–N, and then C–N versus C–O; P–O versus P–F, N–N versus N–O, C–S versus C–Cl. This trend can also be observed for double bonds in C=C versus C=N and C=N versus C=O, and P=O versus S=O. Even for triple bonds, the absolute value of the correlation energy for the C≡C is lower than that of the C≡N bond.

However, we expect a few exceptions, since atomic radii decrease gradually from left to right, but increase more significantly from top to bottom in the periodic table.

Ultimately, the much larger magnitude of the CEPB value in the carbon–bromine bond can be attributed to 3d-block electrons. These unbound electrons do not participate in chemical bonding, but the correlation effects associated with them contribute significantly to the total correlation energy of the molecule, thereby increasing the absolute value of the correlation energy per carbon–bromine bond. As mentioned above, the CEPB method incorporates the core correlation effects into chemical bond fragments, which leads to seemingly anomalous behavior in the case of the carbon–bromine bond.

To show the real predictive power of the CEPB method, we calculated the approximated CCSD­(T) correlation energies for several systems using eq 1 and the CEPB values reported in Table 2.

As an initial evaluation of our method, we calculated correlation energies for the molecules from the training set used to obtain the optimized CEPB values reported in Table 2. In Table 3, we report the CEPB correlation energies calculated in the CBS limit, compared with our reference method, i.e., CCSD­(T), and the CCSD and MP2 results. To assess the quality of the results, mean absolute errors (MAE), mean absolute percentage errors (MAPE), and root mean squared errors (RMS) are computed and presented at the bottom of the table. In the Supporting Information (SI), similar results are presented in Tables S1, S2, and S3 for the aug-cc-pVQZ and aug-cc-pVTZ basis sets (all electron and frozen core), respectively.

Impressively, we can observe that the correlation energies calculated with this simple method are almost of the reference CCSD­(T) quality, with errors smaller than 1% in most cases. The only exceptions are small systems, i.e., methane, ethane, sulfur dioxide, cyclopropyne, and cyclopropenylidiene, for which the errors are slightly greater than 1%. At the same time, the CCSD errors oscillate between 2.74 and 5.61% and the MP2 errors are much larger, i.e., in the range between 1.77 and 11.53%. Analysis of MAE, MAPE, and RMS errors shows that the CEPB method provides significantly better results than the CCSD method. For the molecules from the training set, all errors of the CEPB correlation energies are about one order smaller than those from the CCSD and MP2 methods.

Furthermore, we evaluated the CEPB method’s performance for the *test set* consisting of larger molecules, for which we could calculate the CCSD­(T) energies. Almost all test molecules contain more than ten atoms; the biggest ones are cyclohexene (16 atoms) and cyclohexane (18 atoms). The results are collected in Table 4 for the CBS limit and Tables S4 and S5 in the SI for aug-cc-pVQZ and aug-cc-pVTZ basis sets, respectively. To reduce the computational cost of geometry optimization, we used optimized geometries from the NIST Database^53^ at varying levels of accuracy. All relevant information regarding geometry optimization details is included in Table 4.

**Table 4:** Comparison of the CCSD­(T), CCSD, MP2, and CEPB Correlation Energies (in *E*_h_) for the Set of 18 Molecules Obtained in the CBS Limit. The Percentage Errors Related to the CCSD­(T) Method are Presented in the Last Three Columns. The MAE, RMS (in *E*_h_), and MAPE (in %) are Reported in the Last Rows.

		*E*_*i*_^corr^ (*E*_h_)		error (%)		
compound		CCSD(T)	CEPB	CEPB	CCSD	MP2
uracil(lactam)^c^	C_4_N_2_O_2_H_4_	–**2.045552**	–2.052441	0.34	4.35	3.91
uracil(lactim)^c^	C_4_N_2_O_2_H_4_	–**2.047430**	–2.066019	0.91	4.42	3.83
cyclobutene^f^	C_4_H_6_	–**0.937166**	–0.930239	0.74	4.03	6.59
cyclohexene^d^	C_6_H_10_	–**1.429816**	–1.413573	1.14	3.93	6.70
1,4–cyclohexadiene^d^	C_6_H_8_	–**1.386865**	–1.377145	0.70	4.17	6.52
butadiene^f^	C_4_H_6_	–**0.932053**	–0.929121	0.31	4.12	7.01
benzene^e^	C_6_H_6_	–**1.346083**	–1.340716	0.40	4.59	5.51
acetaldehyde^f^	CH_3_CHO	–**0.791964**	–0.789093	0.36	3.69	5.31
propyne^f^	C_3_H_4_	–**0.687091**	–0.681379	0.83	4.10	6.35
allene[Table-fn t4fn7]	C_3_H_4_	–**0.687131**	–0.687454	0.05	4.20	7.12
cyclohexane[Table-fn t4fn8]	C_6_H_12_	–**1.472888**	–1.450001	1.55	3.67	6.81
cysteine^f^	C_3_H_7_NO_2_S	–**1.931040**	–1.926589	0.23	3.93	4.88
nitrosobenzene^f^	C_6_H_5_NO	–**1.906279**	–1.907898	0.08	4.65	4.60
tetramethyldiphosphine^f^	C_4_H_12_P_2_	–**1.527729**	–1.522725	0.33	3.99	7.64
chlorodimethylsulfonium chloride^f^	C_2_H_6_Cl_2_S	–**1.509093**	–1.513004	0.26	4.31	6.79
nitrosopyrrolidine^f^	C_4_H_8_N_2_O	–**1.828895**	–1.822915	0.33	4.14	4.94
nitrosodiethylamine^f^	C_4_H_10_N_2_O	–**1.869924**	–1.858224	0.63	4.02	5.28
adenine^f^	C_5_H_5_N_5_	–**2.443147**	–2.469458	1.08	4.67	3.83
Level of geometry optimization:			**MAE**	0.009	0.063	0.081
			**MAPE**	0.57	4.17	5.76
			**RMS**	0.011	0.067	0.084

a CCSD/aug-cc-pVDZ.

b QCISD/6-311G*.

c CCSD­(T)/cc-pVTZ.

d CCSD = FULL/cc-pVTZ

e CCSD­(T)=FULL/cc-pVTZ.

f MP2 = FULL/cc-pVTZ.

Even though we utilized geometries from NIST,^53^ the quality of the results remains better than that obtained by the CCSD and MP2 methods. Compared to the CBS limit, CEPB has errors bigger than 1% for three systems: cyclohexene (1.14%), cyclohexane (1.55%), and adenine (1.08%). Again, these errors are about twice as small as those from the CCSD and MP2 methods, showing significant potential capabilities of the CEPB method in correlation energy predictions.

We have also calculated the CEPB correlation energies for a few larger systems for which the CCSD­(T) correlation energies are provided.^74^ Because, in this case, the CCSD­(T) correlation energies were obtained with the frozen core approximation, to calculate the CEPB energies, we use the CEPB values from the last column in Table 2. The results are reported in Table 5. The molecules in the table are arranged into two groups: CEMS26-set, introduced by Kallay et al.,^74^ and NWH-set.^75^

**Table 5:** Comparison of the CEPB (aug-cc-pVTZ, Frozen Core) and CCSD­(T) (Several Basis Sets, Frozen Core) Correlation Energies for the Set of Large Molecules. The Reference Correlation Energies for CCSD­(T), CCSD, and MP2 were Obtained from ref 74. The MAE, RMS (in *E*_h_), and MAPE (in %) are Reported in the Last Rows

			*E*_*i*_^corr^ (*E*_h_)		error (%)		
set	system	atoms	CCSD(T)	CEPB	CEPB	CCSD	MP2
CEMS26	penicillin V	42	**-4.550853** ^d^	–4.655319	2.30	4.72^d^	6.39^d^
	porphyrin	38	–**4.189472** ^b^	–4.220683	0.74	5.69^b^	5.22^b^
	octamethylcyclobutane (OMCB)	36	–**2.374168** ^c^	–2.292621	3.43	4.26^c^	8.67^c^
	deoxycytidylic acid (dCMP)	34	–**4.022583** ^b^	–4.143485	3.01	4.19^b^	5.44^b^
	Pph_3_	34	–**3.219445** [Table-fn t5fn5]	–3.248403	0.90	5.44[Table-fn t5fn5]	7.01[Table-fn t5fn5]
	Gly_4_	31	–**3.523466** ^c^	–3.539575	0.46	4.37^c^	5.66^c^
	diclofenac	30	–**3.459576** ^d^	–3.524700	1.88	5.11^d^	6.62^d^
NWH	[2.2]paracyclophane	32	–**2.841173** ^c^	–2.782671	2.06	5.33^c^	6.68^c^
	*n*-octane	26	–**1.587915** ^c^	–1.560556	1.72	3.85^c^	9.47^c^
	hexanoic acid	20	–**1.645987** ^c^	–1.629577	1.00	4.08^c^	7.29^c^
				**MAE**	0.055	0.151	0.205
				**MAPE**	1.75	4.70	6.85
				**RMS**	0.066	0.161	0.210

a cc-pVTZ.

b aug-cc-pVTZ.

c def2-TZVP.

d cc-pVTZ/density fitting.

One can note, that all error values are similar to the ones of the training (Table 3), and test sets (Table 4), being only slightly bigger. Note, however, that Kallay’s^74^ CCSD­(T) correlation energies in Table 5 are calculated with slightly different basis sets than the CEPB ones (see the captions in Table 5). This again confirms that the CEPB method can successfully predict approximated correlation energies for various reference methods and very large systems with different basis sets.

Next, to investigate a potentially practical utility of this method, we have employed the CEPB energies to calculate the reaction energies of several species. We want to emphasize that, in general, reaction energies are very sensitive quantities. The quality of the obtained results depends strongly on the seemingly insignificant effects, which are almost unnoticeable for total or even correlation energies. This was also to some extent discussed in ref 76. Hence, at the current stage of development of our method, we do not expect extraordinary behavior when calculating reaction energies. This is related to a few aspects. First, we must note that our procedure currently does not distinguish between positional isomers. For example, we cannot differentiate between specific structural isomers, such as pentane-1-ol, pentane-2-ol, and pentane-3-ol, which differ only in the position of the hydroxyl (−OH) group. For these molecules, the CEPB correlation energies are identical. Another limitation is related to the lack of geometry dependence in CEPB values. Lastly, the CEPB method cannot treat systems with no well-defined Lewis structure, such as transition-metal complexes or antiaromatic molecules. However, these types of systems, mainly due to their multireference character, are known to be computationally challenging and cannot be adequately described using single-reference methods such as CCSD­(T).

Keeping that in mind, we have investigated the 22 reaction energies of various types with the CEPB method. Fourteen reactions were taken from Grimme’s work^77^ with two additional Diels–Alder reactions from Zhao and Truhlar’s studies,^78^ and a few reactions from ref 79. These are reported in Table 6 together with CCSD­(T), CCSD, MP2, and HF methods results. In this case, the overall performance of the CEPB method is very similar to that obtained from the MP2 method. However, the results obtained from the CCSD method are much better than the reaction energies from CEPB. Several factors may contribute to the significantly poorer performance of the CEPB method, e.g., omitting the impact of nonvalence electrons or neglecting mutual interaction between neighboring chemical bonds (i.e., not accounting for different environments of the same types of bonds). Here, the term environment refers to the position of a given chemical bond within the molecular structure. A subtle yet noticeable effect of bond positioning can be observed, particularly in the case of positional isomers. Consider again, for instance, the three isomers: 2-methylphenol, 3-methylphenol, and 4-methylphenol. Each of these molecules contains the same number and types of chemical bonds, leading the CEPB method to assign them identical correlation energies. However, accurate quantum chemical computations can reveal very small differences in their correlation energies, which at the current stage of research is not captured by the CEPB method. The sole distinction between these molecules lies in the relative position of hydroxyl and methyl groups, which gives rise to different chemical environments of respective chemical bonds. It is worth mentioning that, from the CEPB perspective, the computation of isomerization energies for these isomers does not yield zero values, but instead collapses to the HF reaction energies. As we mentioned before, the CEPB method, in the simplest initial version presented here, works well for absolute correlation energies but cannot detect more subtle effects revealing themselves in the relative correlation energies. Moreover, the CEPB method shares the same features as CCSD and MP2 counterparts. We observe CEPB results are burdened with relatively large errors when the reaction energy is rather small (e.g., reactions 7 and 9). Our procedure usually provides good-quality results for larger reaction energies similar to those of the counterparts. We emphasize, however, that the computational cost of the CEPB method in the case of reaction energies is as small as that for the HF method; the cost of CEPB is negligible. Hence, our method can efficiently improve the HF results to MP2 quality at almost zero cost. As a final application, we have calculated approximated CCSD­(T) energies using the CEPB method for a few large systems collected in Table 7. The CEPB energies were computed using correlation energies per bond data in CBS limit with full electron correlation (column 3), as well as with aug-cc-pVTZ basis set and frozen core approximation (column 4), using values from Table 2. In that case, we compare the CEPB results with the correlation energies from the literature, where different methods were used. Here, a comparison is made to evaluate their quantitative similarity.

**Table 6:** Reaction Energies (in kcal/mol) Calculated for the Reference CCSD­(T) Method in CBS Limit along with the Deviations of CEPB, CCSD, MP2, and HF Reaction Energies from the CCSD­(T) Reference Values^a^

		*E*_reac_ (kcal/mol)	error (kcal/mol)			
L.p	reaction	CCSD(T)	CEPB	CCSD	MP2	HF
1	HCN + H_2_O → CO + NH_3_	–**11.6**	0.3	–0.8	3.0	–8.7
2	C_2_H_2_ + C_2_H_4_ → cyclobuten	–**31.8**	2.0	0.3	–2.1	7.3
3	ethene + butadiene → cyclohexene	–**46.1**	7.8	–0.5	–5.1	9.2
4	ethyne + butadiene → 1.4–cyclohexadiene	–**61.3**	0.6	–0.5	–2.0	5.8
5	3C_2_H_2_ → benzene	–**152.9**	–4.1	2.2	–5.1	9.4
6	oxirane → CH_3_CHO	–**26.1**	2.5	–0.2	1.1	–4.5
7	CO + CH_4_ → CH_3_CHO	**2.2**	–0.6	0.7	–1.3	9.3
8	cyclopropene → propyne	–**23.8**	1.4	–0.3	–0.3	–3.1
9	HCOOH → CO_2_ + H_2_	**3.0**	0.9	1.2	–2.4	–2.1
10	CO + H_2_O → CO_2_ + H_2_	–**6.3**	–1.5	2.2	–3.5	5.7
11	C_2_H_2_ + H_2_ → C_2_H_4_	–**49.3**	–2.6	–0.9	2.1	–1.7
12	CO + 3H_2_ → CH_4_ + H_2_O	–**66.7**	3.2	–1.5	–1.8	8.6
13	cyclobutene → butadiene	–**8.7**	–2.5	0.4	2.3	–3.2
14	SO_3_ + CO → SO_2_ + CO_2_	–**44.3**	10.4	1.3	–2.5	4.5
15	H_2_O_2_ + H_2_ → 2H_2_O	–**88.2**	2.7	–1.8	–4.3	–4.6
16	CH_3_OH + H_2_S → CH_3_SH + H_2_O	–**11.0**	–0.6	0.5	–1.8	3.4
17	CO + NH_3_ → HCONH_2_	–**8.6**	–3.2	1.0	–2.0	6.5
18	CS_2_ + 2H_2_O → CO_2_ + 2H_2_S	–**10.7**	0.8	–4.6	7.3	–19.9
19	C_2_H_6_ + H_2_ → 2CH_4_	–**18.6**	0.9	–0.6	0.5	–2.7
20	CO + H_2_O_2_ → CO_2_ + H_2_O	–**94.6**	1.3	0.4	–7.8	1.1
21	CH_4_ + 4H_2_O_2_ → CO_2_ + 6H_2_O	–**292.6**	6.5	–3.5	–19.0	–21.3
22	SO_2_ + H_2_O_2_ → SO_3_ + H_2_O	–**50.3**	–9.1	–0.9	–5.3	–3.5
		MAE	3.0	1.2	3.7	6.6
		MAPE	12.1	9.1	19.4	52.2
		RMS	4.1	1.6	5.4	8.4

a The MAE, RMS (in kcal/mol), and MAPE (in %) are reported in the last rows.

**Table 7:** CEPB Correlation Energies (in *E*_h_) Calculated at Two Different Levels (CCSD­(T)/CBS and CCSD­(T)/aug-cc-pVTZ with Frozen Core) for Several Relatively Large Systems with Reference Data in the Last Column (if Available)

	*E*_*i*_^corr^ (*E*_h_)			
		CEPB		literature
system	atoms	CBS	aug-cc-pVTZ (fc)	(if exists)
artemisine (antimalarial)	42	–5.061908	–3.966506	–4.434693^e^
cortisol	56	–6.422682	–5.030026	–5.641822^e^
Buckminsterfullerene C_60_	60	–12.347874	–9.449492	–10.383119^e^
maltotriose	66	–9.343633	–7.323614	–8.186897^e^
cholecalciferol	72	–6.661480	–5.236269	–5.852617^e^
cholesterol	74	–6.699028	–5.274086	–5.911319^e^
atorvastatin	76	–9.869932	–7.681962	–8.432055[Table-fn t7fn6]
vancomycin	176	–25.558242	–19.676399	–19.463164^b^
C_150_H_302_	452	–36.285344	–28.682721	–27.8195^c^
crambin	644	–83.721796	–65.315973	–51.2247^d^

a J. Chem. Phys. 148, 011101 (2018), def2-TZVP/DLPNO–CCSD­(T).

b J. Chem. Phys. 139, 134101 (2013), def2-TZVPP/DLPNO–CCSD­(T).

c J. Chem. Phys. 139, 134101 (2013), def2-SVP/DLPNO–CCSD­(T).

d *E*_UAIQM_GFN2‑xTB*_@CC, ver. 20240106_–*E*_HF/cc‑pV5Z._.

e *E*_UAIQM_GFN2‑xTB*_@CC, ver. 20250115_–*E*_HF/cc‑pV5Z._.

Examination of Table 7 reveals that for vancomycin and C_150_H_302_, the difference between the correlation energies reported in the literature and the aug-cc-pVTZ­(fc) CEPB data remains relatively small. This can easily be justified, as the DLPNO–CCSD­(T) method is known for its robustness and is a computationally efficient alternative to the CCSD­(T) method. In these two cases, the basis sets are sufficiently large and comparable to aug-cc-pVTZ, which we used in our analysis. However, for the crambin molecule, the difference between the reference value and the CEPB-CCSD­(T)/aug-cc-pVTZ­(fc) result is significantly larger, as the def2-SVP basis set is less accurate. This is related to the size of the crambin protein, which is so large that it limits even the application of the DLPNO–CCSD­(T) method to only small basis sets.

Above evaluations demonstrate another possibility of using our method for preliminary estimates of the correlation energies for extensive systems in cases where such values are not available from standard calculations (e.g., at the MP2 level of theory).

Indeed, for many systems presented in Table 7, no literature data is available for the correlation energies due to their extensive size. However, we can use the state-of-the-art UAIQM (here UAIQM_GFN2x‑TB*_@CC, versions 20240106 aka AIQM2 and 20250115) methods,^65^ many of which are approaching coupled-cluster accuracy at the cost of semiempirical quantum mechanical methods. For these systems, the UAIQM correlation energies fall between our CEPB-CCSD­(T)/CBS and CEPB-CCSD­(T)/aug-cc-pVTZ­(fc) variants. It is important to emphasize that the ML-based UAIQM model used here was trained on a data set of small molecules, each containing approximately 10 atoms. Despite this, the quality of computed correlation energies remains satisfactory compared to the CEPB method, which was developed using a completely different methodology. An interesting observation is that UAIQM produced results within just a few seconds, whereas even HF computations lasted many times longer. This is an impressive manifestation of the huge leap in ML-driven computations in quantum chemistry.

## Conclusions

In summary, we introduced a novel CEPB method to estimate electron correlation energies based on the chemical bond concept, combined with high-accuracy quantum-chemical approaches like CCSD­(T). By decomposing molecular correlation energy into bond-specific contributions, i.e., correlation energy per bond, CEPB enables efficient calculations for large molecules, including proteins, while maintaining CCSD­(T) accuracy. We validated its performance across diverse molecular systems of varying sizes, confirming our fundamental assumption and demonstrating the transferability of bond-specific correlation energies. CEPB significantly reduces computational costs for obtaining CCSD­(T)/CBS-quality correlation energies, even for large molecules, without relying on standard approximations such as the frozen core approach. The most computationally demanding step is the initial calculation of CEPB values for a training set of small molecules and bond types using CCSD­(T) method. However, once determined, these bond contributions can be reused across different molecular systems, making the method highly efficient.

At its current stage, CEPB is limited to the simplest and most common types of bonds. Future developments will extend it to additional bond types, including hydrogen bonds, which are crucial for biomolecular systems but pose a greater challenge. Additionally, CEPB does not yet account for noncovalent interactions, an area we aim to explore. We also plan to refine the reference methods, incorporating higher-order electron correlation effects, such as quadruple excitations in CC theory.

Future extensions may include parametrization based on bond lengths and bond orders, which would broaden the applicability of the CEPB method. This enhancement could enable applications such as geometry optimization, vibrational frequency calculations, and more.

One promising application of CEPB is as a benchmarking tool for computational methods like DFT and ML-based approaches, which can efficiently describe correlation effects in large molecules. CEPB provides a computationally feasible reference for such comparisons. However, while it achieves excellent agreement with CCSD­(T) in correlation energy, it does not always ensure gold-standard accuracy for reaction energies, where subtle effects play a decisive role.

Further refinement is necessary to improve isomer differentiation. While CEPB distinguishes functional group isomers and tautomers, it fails to differentiate positional isomers (e.g., butane vs isobutane). This limitation suggests that additional neighborhood effects of chemical bonds must be incorporated, e.g., by including higher-order effects to enhance sensitivity and improve reaction energy predictions.

Also, the correlation effects from inner-shell electrons cannot be neglected, particularly for more electron-rich atoms like sulfur or bromine. It can partially explain why the carbon–bromine bond correlation energy does not adhere to the trends observed for the bonding between different elements with respect to their position in the periodic table; see Figure 4.

In the ML era, there is an increasing need for physically meaningful and computationally efficient molecular descriptors. Our findings demonstrate that the number and types of chemical bonds are critical descriptors for achieving highly accurate correlation energy predictions.

## Supplementary Material



## Notes

Correlation energies for 84 molecules (in *E*_h_) from the training set calculated using CCSD­(T), CCSD, MP2, and CEPB methods in the aug-cc-pVQZ basis set (Table S1); energies also for aug-cc-pVTZ and frozen core aug-cc-pVTZ basis sets, respectively (Tables S2 and S3); contain a comparison of the CCSD­(T), CCSD, MP2, and CEPB correlation energies (in *E*_h_) for the test set of 18 molecules obtained in aug-cc-pVQZ and aug-cc-pVTZ, respectively (Tables S4 and S5) (ZIP)
