# Supplementary material for: Ultrafast Correlation Energy Estimator
Source: J Phys Chem A. 2025 Sep 10;129(38):8877–90. doi: 10.1021/acs.jpca.5c04423 (PMC12478861; doi:10.1021/acs.jpca.5c04423)
Supplement: Supplementary file 1 [file jp5c04423_si_001.pdf]

Supplementary Materials for "Ultrafast correlation energy estimator"

Mateusz Witkowski,[†] Szymon Śmiga,[†] So Hirata,[‡] Pavlo O. Dral,[†,¶,§] and Ireneusz Grabowski[*,†]

†*Institute of Physics, Faculty of Physics, Astronomy, and Informatics, Nicolaus Copernicus University in Toruń, ul. Grudziądzka 5, 87-100 Toruń, Poland*

‡*Department of Chemistry, University of Illinois at Urbana-Champaign, 600 South Mathews Avenue, Urbana, Illinois 61801, USA*

¶*State Key Laboratory of Physical Chemistry of Solid Surfaces, Department of Chemistry, College of Chemistry and Chemical Engineering, and Fujian Provincial Key Laboratory of Theoretical and Computational Chemistry, Xiamen University, Xiamen 361005, China*

§*Aitomistic, Shenzhen 518000, China*

Training set results:

Table S1: Correlation energies for 84 molecules (in E_h) from the *training set* calculated using CCSD(T), CCSD, MP2, and CEPB methods in the aug-cc-pVQZ basis set. The last three columns show the percentage errors with respect to CCSD(T) results. In the last rows, the MAE, RMS (in E_h), and MAPE (in %) are reported.

		E_i^{corr} (E_h)		Error (%)		
**Compound**		**CCSD(T)**	**CEPB**	**CEPB**	**CCSD**	**MP2**
methane	CH_4	**-0.266193**	-0.262663	1.33	2.73	9.73
ethane	C_2H_6	**-0.496465**	-0.491009	1.10	3.11	8.84
ethene	C_2H_4	**-0.453982**	-0.454547	0.12	3.75	8.58
acetylene	C_2H_2	**-0.415608**	-0.411753	0.93	4.44	6.83
formaldehyde	H_2CO	**-0.511392**	-0.510947	0.09	3.75	5.12
carbon monoxide	CO	**-0.460082**	-0.458342	0.38	4.20	4.29
carbon dioxide	CO_2	**-0.758833**	-0.759231	0.05	4.27	2.55
acetonitrile	CH_3CN	**-0.676607**	-0.676047	0.08	4.19	5.76
water	H_2O	**-0.329170**	-0.327687	0.45	2.92	3.79
ammonia	NH_3	**-0.303178**	-0.300846	0.77	3.04	6.31
sulphur dioxide	SO_2	**-0.864187**	-0.852145	1.39	4.68	2.94
sulphur trioxide	SO_3	**-1.154120**	-1.160128	0.52	4.46	2.49
tiirane	C_2H_4S	**-0.727827**	-0.731102	0.45	4.21	7.41
methanethiol	CH_3SH	**-0.530444**	-0.530322	0.02	3.68	8.99
formic acid	$HCOOH$	**-0.802354**	-0.804703	0.29	3.80	3.79
ethanethiol	C_2H_5SH	**-0.763493**	-0.758669	0.63	3.70	8.49
2-mercaptoethanol	C_2H_6OS	**-1.054113**	-1.052425	0.16	3.69	6.74
bromomethane	CH_3Br	**-0.644460**	-0.646299	0.29	3.42	4.56
chloromethane	CH_3Cl	**-0.544678**	-0.547511	0.52	3.59	8.23
dichloromethane	CH_2Cl_2	**-0.827848**	-0.832358	0.54	3.97	7.55
trichloromethane	$CHCl_3$	**-1.114885**	-1.117206	0.21	4.21	7.08
carbon tetrachloride	CCl_4	**-1.404982**	-1.402054	0.21	4.40	6.71
fluoromethane	CH_3F	**-0.563859**	-0.562486	0.24	2.86	5.19
difluoromethane	CH_2F_2	**-0.863126**	-0.862309	0.09	2.95	3.70
trifluoromethane	CHF_3	**-1.162570**	-1.162132	0.04	2.99	2.98
carbon tetrafluoride	CF_4	**-1.460874**	-1.461955	0.07	3.00	2.57
methanol	CH_3OH	**-0.555130**	-0.556419	0.23	3.16	5.85
ethanol	C_2H_5OH	**-0.787074**	-0.784766	0.29	3.31	6.37
thioformaldehyde	H_2CS	**-0.491951**	-0.494184	0.45	4.52	8.48
hydrogen cyanide	HCN	**-0.448126**	-0.447701	0.09	4.50	4.83
carbonyl sulphide	OCS	**-0.741301**	-0.742468	0.16	4.92	4.44
carbon disulphide	CS_2	**-0.727339**	-0.725706	0.22	5.73	6.06
thionyl chloride	$SOCl_2$	**-1.176997**	-1.174157	0.24	4.69	5.74
phosgen	$COCl_2$	**-1.082350**	-1.080642	0.16	4.39	5.49
formyl chloride	$CHClO$	**-0.796174**	-0.795794	0.05	4.20	5.41
thiocyanic acid	$HSCN$	**-0.714376**	-0.715360	0.14	4.66	5.92
isothiocyanic acid	$HNCS$	**-0.719933**	-0.725001	0.70	5.07	5.44
dimethyl ether	C_2H_6O	**-0.784350**	-0.785151	0.10	3.33	6.57

		$E_i^{corr}(E_h)$		Error (%)		
**Compound**		**CCSD(T)**	**CEPB**	**CEPB**	**CCSD**	**MP2**
formamide	$HCONH_2$	**-0.777367**	-0.780639	0.42	3.86	4.58
methylamine	CH_3NH_2	**-0.531454**	-0.532355	0.17	3.25	7.15
hydroxylamine	NH_2OH	**-0.595466**	-0.595926	0.08	3.42	4.82
methanimine	CH_2NH	**-0.490866**	-0.493480	0.53	3.89	6.51
ethenamine	CH_2CHNH_2	**-0.721673**	-0.724239	0.36	3.84	6.80
thioacetaldehyde	CH_3CHS	**-0.723786**	-0.722531	0.17	4.28	8.03
ethenethiol	CH_2CHSH	**-0.722792**	-0.722206	0.08	4.20	8.16
cyclopropene	C_3H_4	**-0.651232**	-0.648577	0.41	4.19	7.02
cyclopropenylidiene	C_3H_2	**-0.587969**	-0.595972	1.36	4.49	7.14
cyclopropyne	C_3H_2	**-0.598618**	-0.605784	1.20	5.10	7.36
sulphur dichloride	SCl_2	**-0.864322**	-0.866174	0.21	4.41	7.86
methoxyamine	CH_5NO	**-0.824103**	-0.824658	0.07	3.51	5.79
N-methylhydroxylamine	CH_5NO	**-0.825933**	-0.827436	0.18	3.51	5.68
chlorosulphuric acid	HSO_3Cl	**-1.473678**	-1.475652	0.13	4.19	3.80
phosphorus trichloride	PCl_3	**-1.118733**	-1.119681	0.08	4.41	7.42
phosphine	PH_3	**-0.272533**	-0.271108	0.52	3.29	12.39
hypophosphorous acid	H_3PO_2	**-0.853976**	-0.855398	0.17	3.61	4.40
phosphoryl chloride	$POCl_3$	**-1.415536**	-1.414588	0.07	4.31	5.80
diphosphane	P_2H_4	**-0.512557**	-0.512499	0.01	3.94	11.19
orthophosphoric acid	H_3PO_4	**-1.434639**	-1.434165	0.03	3.58	3.28
phosphorus trifluoride	PF_3	**-1.174972**	-1.174897	0.01	3.13	3.02
diphosphorus tetrafluoride	P_2F_4	**-1.717494**	-1.717550	0.00	3.37	3.68
diaminomethane	$NH_2CH_2NH_2$	**-0.799400**	-0.802047	0.33	3.50	6.18
hydrazine	N_2H_4	**-0.570353**	-0.569168	0.21	3.48	5.90
hydrogen peroxide	H_2O_2	**-0.625930**	-0.627292	0.22	3.52	3.96
1,1-dimethylhydrazine	$H_2N_2(CH_3)_2$	**-1.033514**	-1.032186	0.13	3.61	6.64
oxirane	H_2COCH_2	**-0.749001**	-0.750835	0.24	3.72	5.61
methyl hydroperoxide	H_3CO_2H	**-0.854868**	-0.856023	0.14	3.60	5.11
hydroperoxyamine	H_2NO_2H	**-0.898048**	-0.895531	0.28	3.84	4.42
methyldisulfide	H_3CS_2H	**-0.798447**	-0.801274	0.35	4.14	8.56
bromoethane	$C2H_5Br$	**-0.878709**	-0.874645	0.46	3.51	5.27
dibromomethane	CH_2Br_2	**-1.028822**	-1.029934	0.11	3.69	3.02
ethanimine	C_2H_5N	**-0.720608**	-0.721826	0.17	3.84	6.95
dimethyldisulfide	$C_2H_6S_2$	**-1.033007**	-1.036369	0.33	4.12	8.19
methyl-disphosphin	CH_6P_2	**-0.746591**	-0.746593	0.00	3.93	9.86
methylphosphine	CH_5P	**-0.504373**	-0.505202	0.16	3.50	10.00
propargylchloroformate	$C_4H_3ClO_2$	**-1.699031**	-1.695720	0.19	4.30	5.05
nitrosamine	H_2N_2O	**-0.824739**	-0.827386	0.32	4.29	4.14
trimethylphosphine	C_3H_9P	**-0.973667**	-0.973390	0.03	3.69	8.51
thioureadioxide	$CH_4N_2O_2S$	**-1.626700**	-1.617799	0.55	4.30	4.35
methylhydrazine	CH_3NHNH_2	**-0.798965**	-0.800677	0.21	3.51	6.42
disulfane	H_2S_2	**-0.564475**	-0.566179	0.30	4.20	9.21
methanesulfonicacid	CH_4O_3S	**-1.418346**	-1.425273	0.49	3.93	4.00
nitrosodimethylamine	$(CH_3)_2NN{=}O$	**-1.292250**	-1.290404	0.14	4.17	5.30
thiothiophthene	$C_5H_4S_3$	**-1.884409**	-1.880462	0.21	5.54	5.88

		$E_i^{corr}(E_h)$		Error (%)		
**Compound**		**CCSD(T)**	**CEPB**	**CEPB**	**CCSD**	**MP2**
nitroxyl	HNO	**-0.559864**	-0.559063	0.14	4.31	4.69
			**MAE**	0.002	0.033	0.048
			**MAPE**	0.31	3.91	6.08
			**RMS**	0.003	0.037	0.051

Table S2: Correlation energies for 84 molecules from the *training set* calculated using CCSD(T), CCSD, MP2, and CEPB methods in the aug-cc-pVTZ basis set. The last three columns show the percentage errors with respect to CCSD(T) results. In the last rows, the MAE, RMS (in E_h), and MAPE (in %) are reported.

		E_i^{corr} (E_h)		Error (%)		
**Compound**		**CCSD(T)**	**CEPB**	**CEPB**	**CCSD**	**MP2**
methane	CH_4	**-0.246639**	-0.243048	1.46	2.72	11.08
ethane	C_2H_6	**-0.458498**	-0.453141	1.17	3.11	10.09
ethene	C_2H_4	**-0.415708**	-0.416631	0.22	3.80	9.81
acetylene	C_2H_2	**-0.377116**	-0.373439	0.98	4.56	7.88
formaldehyde	H_2CO	**-0.461259**	-0.460956	0.07	3.82	5.95
carbon monoxide	CO	**-0.410026**	-0.406284	0.91	4.35	5.00
carbon dioxide	CO_2	**-0.678502**	-0.678865	0.05	4.43	2.99
acetonitrile	CH_3CN	**-0.615071**	-0.614304	0.12	4.28	6.68
water	H_2O	**-0.296828**	-0.295397	0.48	2.95	4.51
ammonia	NH_3	**-0.277412**	-0.274582	1.02	3.05	7.35
sulphur dioxide	SO_2	**-0.766166**	-0.753568	1.64	4.83	3.43
sulphur trioxide	SO_3	**-1.023619**	-1.030076	0.63	4.59	2.89
tiirane	C_2H_4S	**-0.657218**	-0.661630	0.67	4.23	8.59
methanethiol	CH_3SH	**-0.478054**	-0.477661	0.08	3.66	10.37
formic acid	$HCOOH$	**-0.720865**	-0.723712	0.39	3.89	4.44
ethanethiol	C_2H_5SH	**-0.693461**	-0.687754	0.82	3.70	9.76
2-mercaptoethanol	C_2H_6OS	**-0.953376**	-0.950510	0.30	3.71	7.81
bromomethane	CH_3Br	**-0.528164**	-0.530118	0.37	3.57	7.01
chloromethane	CH_3Cl	**-0.480771**	-0.483667	0.60	3.58	9.53
dichloromethane	CH_2Cl_2	**-0.719944**	-0.724287	0.60	3.98	8.77
trichloromethane	$CHCl_3$	**-0.962956**	-0.964906	0.20	4.25	8.23
carbon tetrachloride	CCl_4	**-1.208509**	-1.205526	0.25	4.45	7.80
fluoromethane	CH_3F	**-0.506757**	-0.505281	0.29	2.89	6.02
difluoromethane	CH_2F_2	**-0.768409**	-0.767514	0.12	3.00	4.30
trifluoromethane	CHF_3	**-1.030201**	-1.029748	0.04	3.05	3.43
carbon tetrafluoride	CF_4	**-1.290825**	-1.291981	0.09	3.07	2.95
methanol	CH_3OH	**-0.504301**	-0.505803	0.30	3.19	6.80
ethanol	C_2H_5OH	**-0.718109**	-0.715896	0.31	3.34	7.37
thioformaldehyde	H_2CS	**-0.439099**	-0.440802	0.39	4.58	9.85
hydrogen cyanide	HCN	**-0.404940**	-0.404211	0.18	4.63	5.65
carbonyl sulphide	OCS	**-0.657239**	-0.658710	0.22	5.09	5.19
carbon disulphide	CS_2	**-0.639462**	-0.638556	0.14	5.94	7.12
thionyl chloride	$SOCl_2$	**-1.020007**	-1.016745	0.32	4.79	6.64

		$E_i^{corr}(E_h)$		Error (%)		
**Compound**		**CCSD(T)**	**CEPB**	**CEPB**	**CCSD**	**MP2**
phosgen	$COCl_2$	**-0.942747**	-0.942196	0.06	4.49	6.34
formyl chloride	$CHClO$	**-0.701512**	-0.701576	0.01	4.29	6.26
thiocyanic acid	$HSCN$	**-0.637328**	-0.638824	0.23	4.77	6.88
isothiocyanic acid	$HNCS$	**-0.641883**	-0.647337	0.85	5.23	6.35
dimethyl ether	C_2H_6O	**-0.715172**	-0.716209	0.14	3.35	7.58
formamide	$HCONH_2$	**-0.702054**	-0.706004	0.56	3.94	5.35
methylamine	CH_3NH_2	**-0.487087**	-0.488095	0.21	3.27	8.25
hydroxylamine	NH_2OH	**-0.538602**	-0.539130	0.10	3.47	5.65
methanimine	CH_2NH	**-0.447056**	-0.449582	0.57	3.96	7.53
ethenamine	CH_2CHNH_2	**-0.658741**	-0.661678	0.45	3.90	7.84
thioacetaldehyde	CH_3CHS	**-0.652954**	-0.650895	0.32	4.33	9.28
ethenethiol	CH_2CHSH	**-0.652044**	-0.651244	0.12	4.24	9.39
cyclopropene	C_3H_4	**-0.595222**	-0.593770	0.24	4.26	8.07
cyclopropenylidiene	C_3H_2	**-0.531195**	-0.539097	1.49	4.62	8.22
cyclopropyne	C_3H_2	**-0.542437**	-0.550578	1.50	5.27	8.47
sulphur dichloride	SCl_2	**-0.737875**	-0.740238	0.32	4.44	9.20
methoxyamine	CH_5NO	**-0.748912**	-0.749535	0.08	3.55	6.73
N-methylhydroxylamine	CH_5NO	**-0.751056**	-0.752642	0.21	3.55	6.61
chlorosulphuric acid	HSO_3Cl	**-1.299162**	-1.300959	0.14	4.27	4.39
phosphorus trichloride	PCl_3	**-0.965473**	-0.966975	0.16	4.41	8.54
phosphine	PH_3	**-0.257377**	-0.255519	0.72	3.15	13.67
hypophosphorous acid	H_3PO_2	**-0.774002**	-0.776254	0.29	3.62	5.05
phosphoryl chloride	$POCl_3$	**-1.231963**	-1.230461	0.12	4.34	6.61
diphosphane	P_2H_4	**-0.482271**	-0.482403	0.03	3.80	12.38
orthophosphoric acid	H_3PO_4	**-1.291502**	-1.290751	0.06	3.62	3.80
phosphorus trifluoride	PF_3	**-1.046057**	-1.045987	0.01	3.17	3.43
diphosphorus tetrafluoride	P_2F_4	**-1.536308**	-1.536360	0.00	3.41	4.18
diaminomethane	$NH_2CH_2NH_2$	**-0.730636**	-0.733142	0.34	3.53	7.17
hydrazine	N_2H_4	**-0.519914**	-0.518555	0.26	3.52	6.88
hydrogen peroxide	H_2O_2	**-0.562925**	-0.564402	0.26	3.58	4.67
1,1-dimethylhydrazine	$H_2N_2(CH_3)_2$	**-0.947068**	-0.945581	0.16	3.64	7.67
oxirane	H_2COCH_2	**-0.680404**	-0.683254	0.42	3.78	6.51
methyl hydroperoxide	H_3CO_2H	**-0.773549**	-0.774808	0.16	3.65	5.96
hydroperoxyamine	H_2NO_2H	**-0.810871**	-0.808135	0.34	3.92	5.19
methyldisulfide	H_3CS_2H	**-0.711722**	-0.715117	0.48	4.13	9.92
bromoethane	$C2H_5Br$	**-0.744820**	-0.740211	0.62	3.63	7.38
dibromomethane	CH_2Br_2	**-0.815861**	-0.817189	0.16	3.93	5.51
ethanimine	C_2H_5N	**-0.658568**	-0.659676	0.17	3.89	8.01
dimethyldisulfide	$C_2H_6S_2$	**-0.928362**	-0.932040	0.40	4.12	9.46
methyl-disphosphin	CH_6P_2	**-0.698906**	-0.698720	0.03	3.84	11.00
methylphosphine	CH_5P	**-0.471290**	-0.471836	0.12	3.43	11.21
propargylchloroformate	$C_4H_3ClO_2$	**-1.519685**	-1.515222	0.29	4.40	5.84
nitrosamine	H_2N_2O	**-0.744469**	-0.747601	0.42	4.40	4.85
trimethylphosphine	C_3H_9P	**-0.904591**	-0.904471	0.01	3.66	9.63
thioureadioxide	$CH_4N_2O_2S$	**-1.462294**	-1.453206	0.62	4.39	5.07
methylhydrazine	CH_3NHNH_2	**-0.730211**	-0.732068	0.25	3.54	7.44

Compound		$E_i^{corr}(E_h)$		Error (%)		
		**CCSD(T)**	**CEPB**	**CEPB**	**CCSD**	**MP2**
disulfane	H_2S_2	**-0.495757**	-0.498193	0.49	4.17	10.75
methanesulfonicacid	CH_4O_3S	**-1.270841**	-1.278132	0.57	4.00	4.66
nitrosodimethylamine	$(CH_3)_2NN{=}O$	**-1.176770**	-1.174626	0.18	4.25	6.16
thiothiophthene	$C_5H_4S_3$	**-1.695756**	-1.691002	0.28	5.68	6.84
nitroxyl	HNO	**-0.504616**	-0.503628	0.20	4.43	5.50
			**MAE**	0.002	0.030	0.050
			**MAPE**	0.38	3.97	7.08
			**RMS**	0.003	0.033	0.053

Table S3: Correlation energies for 84 molecules (in E_h) from the *training set* calculated using CCSD(T), CCSD, MP2, and CEPB methods in the aug-cc-pVTZ basis set with frozen core approximation. The last three columns show the percentage errors with respect to CCSD(T) results. In the last rows, the MAE, RMS (in E_h), and MAPE (in %) are reported.

Compound		E_i^{corr} (E_h)		Error (%)		
		**CCSD(T)**	**CEPB**	**CEPB**	**CCSD**	**MP2**
methane	CH_4	**-0.227173**	-0.223193	1.75	2.87	11.62
ethane	C_2H_6	**-0.419314**	-0.414245	1.21	3.31	10.59
ethene	C_2H_4	**-0.378479**	-0.379266	0.21	4.06	10.32
acetylene	C_2H_2	**-0.341366**	-0.337887	1.02	4.91	8.26
formaldehyde	H_2CO	**-0.428206**	-0.427227	0.23	4.03	6.20
carbon monoxide	CO	**-0.380216**	-0.373654	1.73	4.59	5.24
carbon dioxide	CO_2	**-0.629889**	-0.631261	0.22	4.68	3.06
acetonitrile	CH_3CN	**-0.561185**	-0.561588	0.07	4.58	6.96
water	H_2O	**-0.281576**	-0.280132	0.51	3.06	4.73
ammonia	NH_3	**-0.260037**	-0.257561	0.95	3.19	7.68
sulphur dioxide	SO_2	**-0.698638**	-0.688102	1.51	5.12	3.82
sulphur trioxide	SO_3	**-0.939427**	-0.945118	0.61	4.85	3.15
tiirane	C_2H_4S	**-0.579841**	-0.583508	0.63	4.58	9.57
methanethiol	CH_3SH	**-0.419736**	-0.418710	0.24	3.92	11.74
formic acid	HCOOH	**-0.671956**	-0.674318	0.35	4.10	4.62
ethanethiol	C_2H_5SH	**-0.614573**	-0.609762	0.78	3.97	10.82
2-mercaptoethanol	C_2H_6OS	**-0.858575**	-0.856853	0.20	3.95	8.52
bromomethane	CH_3Br	**-0.461443**	-0.462820	0.30	3.69	8.64
chloromethane	CH_3Cl	**-0.426391**	-0.428900	0.59	3.82	10.81
dichloromethane	CH_2Cl_2	**-0.630374**	-0.634607	0.67	4.28	10.26
trichloromethane	$CHCl_3$	**-0.838252**	-0.840314	0.25	4.58	9.80
carbon tetrachloride	CCl_4	**-1.049218**	-1.046020	0.30	4.81	9.39
fluoromethane	CH_3F	**-0.473351**	-0.471377	0.42	3.03	6.27
difluoromethane	CH_2F_2	**-0.720844**	-0.719562	0.18	3.14	4.48
trifluoromethane	CHF_3	**-0.968275**	-0.967746	0.05	3.19	3.59
carbon tetrafluoride	CF_4	**-1.214398**	-1.215930	0.13	3.20	3.09
methanol	CH_3OH	**-0.469605**	-0.470285	0.14	3.35	7.09
ethanol	C_2H_5OH	**-0.663262**	-0.661336	0.29	3.53	7.69

		$E_i^{corr}(E_h)$		Error (%)		
**Compound**		**CCSD(T)**	**CEPB**	**CEPB**	**CCSD**	**MP2**
thioformaldehyde	H_2CS	**-0.383657**	-0.385352	0.44	4.95	11.25
hydrogen cyanide	HCN	**-0.371254**	-0.370536	0.19	4.93	5.86
carbonyl sulphide	OCS	**-0.587920**	-0.589386	0.25	5.47	5.80
carbon disulphide	CS_2	**-0.549026**	-0.547510	0.28	6.55	8.47
thionyl chloride	$SOCl_2$	**-0.896832**	-0.892322	0.50	5.15	7.87
phosgen	$COCl_2$	**-0.839965**	-0.838641	0.16	4.80	7.31
formyl chloride	CHClO	**-0.633436**	-0.632934	0.08	4.56	6.98
thiocyanic acid	HSCN	**-0.565738**	-0.566053	0.06	5.15	7.69
isothiocyanic acid	HNCS	**-0.570181**	-0.575396	0.91	5.66	7.09
dimethyl ether	C_2H_6O	**-0.660821**	-0.660437	0.06	3.55	7.91
formamide	$HCONH_2$	**-0.650652**	-0.654663	0.62	4.17	5.56
methylamine	CH_3NH_2	**-0.450200**	-0.450629	0.10	3.46	8.63
hydroxylamine	NH_2OH	**-0.506496**	-0.507182	0.14	3.62	5.93
methanimine	CH_2NH	**-0.411000**	-0.413237	0.54	4.20	7.89
ethenamine	CH_2CHNH_2	**-0.603355**	-0.606702	0.55	4.16	8.21
thioacetaldehyde	CH_3CHS	**-0.576804**	-0.576404	0.07	4.68	10.33
ethenethiol	CH_2CHSH	**-0.575099**	-0.574783	0.06	4.58	10.47
cyclopropene	C_3H_4	**-0.538380**	-0.538176	0.04	4.59	8.46
cyclopropenylidiene	C_3H_2	**-0.478332**	-0.484603	1.31	5.00	8.70
cyclopropyne	C_3H_2	**-0.492102**	-0.496798	0.95	5.68	8.96
sulphur dichloride	SCl_2	**-0.631847**	-0.635306	0.55	4.79	11.23
methoxyamine	CH_5NO	**-0.697243**	-0.697334	0.01	3.73	7.03
N-methylhydroxylamine	CH_5NO	**-0.698906**	-0.700250	0.19	3.74	6.91
chlorosulphuric acid	HSO_3Cl	**-1.176100**	-1.178199	0.18	4.53	4.94
phosphorus trichloride	PCl_3	**-0.813543**	-0.814428	0.11	4.82	10.56
phosphine	PH_3	**-0.208362**	-0.206799	0.75	3.41	16.77
hypophosphorous acid	H_3PO_2	**-0.692270**	-0.693599	0.19	3.86	5.54
phosphoryl chloride	$POCl_3$	**-1.060963**	-1.060078	0.08	4.70	7.97
diphosphane	P_2H_4	**-0.384561**	-0.384675	0.03	4.22	15.42
orthophosphoric acid	H_3PO_4	**-1.176342**	-1.175899	0.04	3.84	4.08
phosphorus trifluoride	PF_3	**-0.953367**	-0.953649	0.03	3.33	3.74
diphosphorus tetrafluoride	P_2F_4	**-1.380687**	-1.380475	0.02	3.60	4.63
diaminomethane	$NH_2CH_2NH_2$	**-0.675775**	-0.678065	0.34	3.74	7.50
hydrazine	N_2H_4	**-0.485600**	-0.484317	0.26	3.69	7.20
hydrogen peroxide	H_2O_2	**-0.533213**	-0.534675	0.27	3.72	4.91
1,1-dimethylhydrazine	$H_2N_2(CH_3)_2$	**-0.872190**	-0.870453	0.20	3.86	8.02
oxirane	H_2COCH_2	**-0.626768**	-0.628296	0.24	4.01	6.78
methyl hydroperoxide	H_3CO_2H	**-0.724169**	-0.724828	0.09	3.83	6.22
hydroperoxyamine	H_2NO_2H	**-0.763846**	-0.761725	0.28	4.09	5.44
methyldisulfide	H_3CS_2H	**-0.614731**	-0.618237	0.57	4.47	11.54
bromoethane	$C2H_5Br$	**-0.657024**	-0.653871	0.48	3.80	8.67
dibromomethane	CH_2Br_2	**-0.701558**	-0.702446	0.13	4.07	7.36
ethanimine	C_2H_5N	**-0.603231**	-0.604289	0.18	4.15	8.38
dimethyldisulfide	$C_2H_6S_2$	**-0.810824**	-0.815176	0.54	4.45	10.77
methyl-disphosphin	CH_6P_2	**-0.580264**	-0.580362	0.02	4.23	13.04
methylphosphine	CH_5P	**-0.401846**	-0.402487	0.16	3.72	12.87

		$E_i^{corr}(E_h)$		Error (%)		
**Compound**		**CCSD(T)**	**CEPB**	**CEPB**	**CCSD**	**MP2**
propargylchloroformate	$C_4H_3ClO_2$	**-1.377141**	-1.375924	0.09	4.72	6.28
nitrosamine	H_2N_2O	**-0.695589**	-0.698654	0.44	4.63	5.08
trimethylphosphine	C_3H_9P	**-0.794108**	-0.793862	0.03	3.97	10.61
thioureadioxide	$CH_4N_2O_2S$	**-1.337343**	-1.328834	0.64	4.67	5.44
methylhydrazine	CH_3NHNH_2	**-0.675745**	-0.677385	0.24	3.74	7.78
disulfane	H_2S_2	**-0.419270**	-0.421297	0.48	4.52	13.01
methanesulfonicacid	CH_4O_3S	**-1.161960**	-1.168370	0.55	4.24	5.01
nitrosodimethylamine	$(CH_3)_2NN{=}O$	**-1.086473**	-1.084790	0.15	4.51	6.42
thiothiophthene	$C_5H_4S_3$	**-1.478789**	-1.473847	0.33	6.25	7.70
nitroxyl	HNO	**-0.473280**	-0.471898	0.29	4.64	5.79
			**MAE**	0.002	0.029	0.049
			**MAPE**	0.38	4.23	7.83
			**RMS**	0.003	0.032	0.053

Test set results:

Table S4: Comparison of the CCSD(T), CCSD, MP2, and CEPB correlation energies (in E_h) for the set of 18 molecules obtained in aug-cc-pVQZ basis set. In the last three columns, the percentage errors related to the CCSD(T) method are presented. In the last rows, the MAE, RMS (in E_h), and MAPE (in %) are reported.

		E_i^{corr} (E_h)		Error (%)		
**Compound**		**CCSD(T)**	**CEPB**	**CEPB**	**CCSD**	**MP2**
uracil(lactam)[a]	$C_4N_2O_2H_4$	**-1.912267**	-1.919201	0.36	4.43	4.34
uracil(lactim)[a]	$C_4N_2O_2H_4$	**-1.914431**	-1.932396	0.94	4.50	4.27
cyclobutene[d]	C_4H_6	**-0.883590**	-0.876924	0.75	4.06	7.26
cyclohexene[b]	C_6H_{10}	**-1.349546**	-1.333618	1.18	3.91	7.31
1,4-cyclohexadiene[b]	C_6H_8	**-1.305400**	-1.297155	0.63	4.16	7.12
butadiene[d]	C_4H_6	**-0.877860**	-0.874777	0.35	4.16	7.71
benzene[c]	C_6H_6	**-1.265574**	-1.260692	0.39	4.65	6.09
acetaldehyde[d]	CH_3CHO	**-0.742002**	-0.739293	0.37	3.73	5.89
propyne[d]	C_3H_4	**-0.645657**	-0.640100	0.86	4.15	7.01
allene[e]	C_3H_4	**-0.646625**	-0.646430	0.03	4.25	7.83
cyclohexane[f]	C_6H_{12}	**-1.394873**	-1.370081	1.78	3.68	7.50
cysteine[d]	$C_3H_7NO_2S$	**-1.805386**	-1.798748	0.37	3.97	5.43
nitrosobenzene[d]	C_6H_5NO	**-1.787679**	-1.788602	0.05	4.72	5.10
tetrametyldiphosphine[d]	$C_4H_{12}P_2$	**-1.454882**	-1.448874	0.41	3.96	8.33
chlorodimethylsulfoniumchloride[d]	$C_2H_6Cl_2S$	**-1.392425**	-1.395413	0.21	4.33	7.48
nitrosopyrrolidine[d]	$C_4H_8N_2O$	**-1.720040**	-1.712781	0.42	4.19	5.49
nitrosodiethylamine[d]	$C_4H_{10}N_2O$	**-1.760168**	-1.747097	0.74	4.07	5.86
adenine[d]	$C_5H_5N_5$	**-2.291125**	-2.315440	1.06	4.75	4.28
Optimization level of geometries:			**MAE**	0.009	0.059	0.084
[a]CCSD/aug-cc-pvdz			**MAPE**	0.61	4.20	6.35
[b]QCISD/6-311G*			**RMS**	0.011	0.064	0.087

[c]CCSD(T)/cc-pvtz

[d]CCSD=FULL/cc-pVTZ

[e]CCSD(T)=FULL/cc-pVTZ

[f]MP2=FULL/cc-pVTZ

Table S5: Comparison of the CCSD(T), CCSD, MP2, and CEPB correlation energies (in E_h) for the set of 18 molecules obtained in aug-cc-pVTZ basis set. In the last three columns, the percentage errors related to the CCSD(T) method are presented. In the last rows, the MAE, RMS (in E_h), and MAPE (in %) are reported.

		E_i^{corr} (E_h)		Error (%)		
**Compound**		**CCSD(T)**	**CEPB**	**CEPB**	**CCSD**	**MP2**
uracil(lactam)[a]	$C_4N_2O_2H_4$	**-1.729618**	-1.736614	0.40	4.55	5.06
uracil(lactim)[a]	$C_4N_2O_2H_4$	**-1.732171**	-1.749282	0.99	4.62	4.97
cyclobutene[d]	C_4H_6	**-0.810170**	-0.803863	0.78	4.12	8.33
cyclohexene[b]	C_6H_{10}	**-1.241263**	-1.224049	1.39	3.95	8.38
1,4-cyclohexadiene[b]	C_6H_8	**-1.196354**	-1.187539	0.74	4.22	8.16
butadiene[d]	C_4H_6	**-0.803595**	-0.800307	0.41	4.23	8.83
benzene[c]	C_6H_6	**-1.155246**	-1.151029	0.37	4.76	7.01
acetaldehyde[d]	CH_3CHO	**-0.673536**	-0.671050	0.37	3.79	6.82
propyne[d]	C_3H_4	**-0.588877**	-0.583532	0.91	4.23	8.07
allene[e]	C_3H_4	**-0.591117**	-0.590214	0.15	4.33	8.96
cyclohexane[f]	C_6H_{12}	**-1.287964**	-1.260560	2.13	3.70	8.59
cysteine[d]	$C_3H_7NO_2S$	**-1.633195**	-1.623559	0.59	4.03	6.30
nitrosobenzene[d]	C_6H_5NO	**-1.625152**	-1.625122	0.00	4.84	5.92
tetrametyldiphosphine[d]	$C_4H_{12}P_2$	**-1.355054**	-1.347672	0.54	3.93	9.40
chlorodimethylsulfoniumchloride[d]	$C_2H_6Cl_2S$	**-1.232547**	-1.234269	0.14	4.37	8.65
nitrosopyrrolidine[d]	$C_4H_8N_2O$	**-1.570869**	-1.561858	0.57	4.27	6.37
nitrosodiethylamine[d]	$C_4H_{10}N_2O$	**-1.609761**	-1.594813	0.93	4.13	6.79
adenine[d]	$C_5H_5N_5$	**-2.082797**	-2.104378	1.04	4.87	5.00
Optimization level of geometries:			**MAE**	0.009	0.055	0.088
[a]CCSD/aug-cc-pvdz			**MAPE**	0.69	4.27	7.31
[b]QCISD/6-311G*			**RMS**	0.012	0.059	0.092

[c]CCSD(T)/cc-pvtz

[d]CCSD=FULL/cc-pVTZ

[e]CCSD(T)=FULL/cc-pVTZ

[f]MP2=FULL/cc-pVTZ
